# Residential Garden Produce Harvested Near a Fluorochemical Manufacturer in North Carolina Can Be An Important Fluoroether Exposure Pathway

**DOI:** 10.1021/acs.jafc.4c06177

**Published:** 2024-11-20

**Authors:** Pingping Meng, Nadia Sheppard, Sarangi Joseph, Owen W. Duckworth, Christopher P. Higgins, Detlef R. U. Knappe

**Affiliations:** † Department of Chemistry, East Carolina University, Greenville, North Carolina 27858, United States; ‡ Department of Civil, Construction, and Environmental Engineering, North Carolina State University, Raleigh, North Carolina 27695, United States; § Department of Crop and Soil Sciences, North Carolina State University, Raleigh, North Carolina 27695, United States; ∥ Department of Civil and Environmental Engineering, Colorado School of Mines, Golden, Colorado 80401, United States; ¶ Center for Human Health and the Environment, North Carolina State University, Raleigh, North Carolina 27695, United States

**Keywords:** fluoroethers, GenX chemicals, air emissions, fruits, vegetables, human exposure

## Abstract

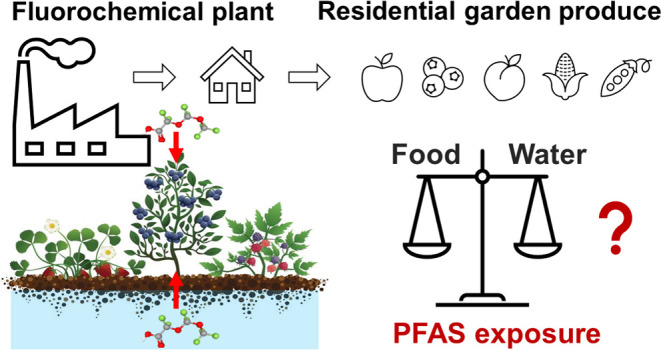

Dietary intake can be an important exposure route to per- and polyfluoroalkyl substances (PFASs). Little is known about the bioaccumulation of emerging per- and polyfluoroalkyl ether acids (PFEAs) in garden produce from PFAS-impacted communities and the associated dietary exposure risk. In this study, 53 produce samples were collected from five residential gardens near a fluorochemical manufacturer. Summed PFAS concentrations ranged from 0.0026 to 38 ng/g wet weight of produce, and water-rich produce exhibited the highest PFAS levels. The PFAS signature was dominated by PFEAs, and hexafluoropropylene oxide-dimer acid (commonly known as GenX) was detected in 72% of samples. Based on average measured GenX concentrations, chronic-exposure daily limits were as low as 289 g produce/day for children (3–6 yr). This analysis does not consider other PFEAs that were present at higher concentrations, but for which reference doses were not available. This study revealed that consuming residential garden produce grown in PFAS-impacted communities can be an important exposure pathway.

## Introduction

Per- and polyfluoroalkyl substances (PFASs) have been widely used in different industries and products since the 1960s, including aqueous film-forming foams (AFFFs), textile and paper coatings, lithium batteries, and nonstick cookware.^1−4^ The widespread use and persistence of PFASs has led to their ubiquitous detection in the environment, with numerous adverse impacts on the environment and human health.^5,6^ The phaseout of long-chain PFASs, such as perfluorooctanesulfonic acid (PFOS) and perfluorooctanesulfonic acid (PFOA), led manufacturers to gradually transition to replacement chemicals with shorter chain lengths and/or different functional groups.^7,8^ Perfluoroalkyl ether acids (PFEAs) are a subclass of PFASs with one or more ether oxygen in the fluorocarbon backbone that are widely produced as replacements for long-chain PFASs or generated as byproducts.^9,10^ For example, hexafluoropropylene oxide-dimer acid (HFPO–DA, commonly known as GenX), has been used as a replacement for PFOA in fluoropolymer manufacturing.^10,11^ Consequently, GenX has been detected with increasing frequency at elevated concentration around the world, including the U.S., Netherlands, Germany, and China.^12−14^ The U.S. Environmental Protection Agency (U.S. EPA) recently finalized a maximum contaminant level (MCL) for Gen X in drinking water at 10 ng/L.^15^

In North Carolina, multiple PFEAs (structures shown in Figure S1), including GenX, are receiving increased public and regulatory attention with their wide detection in private wells and public water systems.^16^ In 2015, 12 PFEAs were identified in North Carolina surface water using a time-of-flight mass spectrometer.^17^ Subsequently, elevated concentrations of multiple PFEAs, including GenX, were found in the lower Cape Fear River watershed in 2016, and PFEAs dominated the PFAS signature in the drinking water of the City of Wilmington, NC.^16^ A fluorochemical manufacturing plant, Fayetteville Works, located ∼150 km upstream on the Cape Fear River near Fayetteville, NC, was identified as the PFEA source.^18^ To address concerns over GenX and other fluoroethers, the North Carolina Department of Environmental Quality (NC DEQ) and Department of Health and Human Service (NC DHHS) began investigating GenX in 2017 and found high concentrations of PFASs in private well water, as well as rainwater, in the area surrounding the fluorochemical manufacturing plant.^19^

In the impacted region of the Cape Fear River basin, PFEAs have been detected not only in water, but also in soil, air, fish, domestic animals, and human blood serum.^20−24^ Three PFEAs—perfluoro-2-{[perfluoro-3-(perfluoroethoxy)-2-propanyl]oxy}ethanesulfonic acid (Nafion byproduct 2), perfluoro-3,5,7,9-butaoxadecanoic acid (PFO4DA), and perfluoro-3,5,7,9,11-pentaoxadodecanoic acid (PFO5DoA)—were detected in 99% of serum samples from people living in Wilmington, North Carolina, about 150 km downstream of the fluorochemical manufacturing plant.^25,26^ The sum concentration of fluoroethers accounted for ∼45% of total measured PFAS burden in serum.^25,26^ Several PFEAs, such as perfluorodioxahexanoic acid (PFO2HxA), PFO4OA, PFO5DoA, and Nafion byproduct 2, were also detected in serum samples from people living in the private well community near the fluorochemical plant.^27^ Such PFAS profiles likely differ from those observed in the general U.S. population although it needs to be noted that, apart from GenX, the PFEAs studied here are typically not targeted by standard analytical methods for PFAS. Additionally, serum concentrations of legacy PFAS in the Wilmington cohort were found to exceed the national averages reported in the 2015–2016 National Health and Nutrition Examination Survey (NHANES).^25^ To date, drinking water has been identified as the major source of PFEA exposure for people living in Wilmington, NC, and the duration of drinking water exposure was associated with higher serum PFAS levels.^28^ This finding agrees with previous studies that compared human daily PFAS intake through different exposure media, and concluded that drinking water exposure is dominant for populations near sources of contaminated drinking water, while food intake is the major exposure pathway for the background population.^29,30^ However, the paucity of reliable concentration data in food and historical exposure data limit the certainty of this assertion.

PFAS exposure through food uptake has been found in other PFAS-impacted populations, mostly focused on fish, livestock, dairy products, and grains.^31−34^ In the lower Cape Fear River basin, a fish consumption advisory has been issued by the NC DHHS, which limits the consumption of some local freshwater fish.^35^ To date, little attention has been paid to noncommercial produce grown in contaminated communities, such as fruits and vegetables. Although the enrichment factors of PFASs in edible plants were much smaller compared to those in fish and meat, researchers found produce, such as lettuce and strawberry, can contain relatively high PFAS concentrations, especially for low molecular weight PFASs.^36^ In communities surrounding the fluorochemical manufacturing plant in Fayetteville, NC, low molecular weight PFEAs were detected as the dominant PFASs in private wells, potentially due to their greater mobility in soils and groundwater.^27^ This may have a strong impact on backyard garden produce, as low molecular weight PFEAs may be readily bioavailable for plant uptake. Residential garden produce was an important part of some residents’ diet before the GenX contamination became widely known in the region. Additionally, high levels of PFEAs in rainwater suggested atmospheric deposition in the region, which may also impact the locally grown produce.^19^ In communities close to fluorochemical manufacturing plants, PFEA exposure through consuming impacted garden produce may be important but remains understudied.

According to the Exposure Factors Handbook developed by the U.S. EPA, the recommended average daily intake of fruits and vegetables in the U.S. is 10.1 g/kg body weight for children aged 3–6 years (equivalent to 186 g/day) and 3.6 g/kg body weight for adults aged 21–50 years (equivalent to 288 g/day).^37^ Neglecting PFAS exposure through potentially contaminated fruits and vegetables may underestimate human exposure risks. To the best of our knowledge, only one relevant study has comprehensively assessed fluoroether exposure pathways near a fluorochemical industrial park in China, finding that gastrointestinal uptake accounted for 99% of PFAS exposure.^38^ However, the previous study focused on one fluoroether, perfluoro-2-methoxyacetic acid (PFMOAA), which exhibited unexpected accumulation in blood serum of the local population. In contrast, this study evaluated a range of structurally distinct PFEAs that have been widely detected in the region surrounding the Fayetteville Works fluorochemical manufacturing plant in North Carolina. While previous studies have examined exposure through drinking water,^27^ the contribution of food—particularly homegrown produce—to PFEA exposure remains unexplored. The overarching goal of this study was to (1) quantify the concentration of PFASs in residential garden produce in an impacted community close to a fluorochemical manufacturer; (2) investigate the temporal and spatial trend of PFAS contamination in produce; and (3) assess human exposure through residential garden produce in a PFAS-impacted community.

## Materials and Methods

### Materials

Forty-three PFASs were targeted (Table S1) in this study, including 11 perfluoroalkyl carboxylic acids (PFCAs), 7 perfluoroalkyl sulfonic acids (PFSAs), 10 per- and polyfluoroalkyl ether carboxylic acids (PFECAs), 3 per- and polyfluoroalkyl ether sulfonic acids (PFESAs), 3 fluorotelomer sulfonic acids (FTSs), 4 fluorotelomer (unsaturated) carboxylic acids (FTCAs and FTUCAs), 3 perfluoroalkane sulfonamides (FASAs), and 2 perfluoroalkane sulfonamido acetic acids (FASAAs). Native PFAS standards were obtained from Wellington Laboratories (Guelph, ON), Fluoryx Laboratories (Carson City, NV), and the Chemours Company (Wilmington, DE) as shown in Table S1. Twenty-four isotopically labeled PFASs were purchased from Wellington Laboratories (Guelph, ON), as shown in Table S2. Methanol (LC-MS grade, Honeywell Burdick & Jackson), ammonium hydroxide (Certified ACS Plus, Fisher Chemical) and ammonium acetate (LC-MS grade, Optima) were purchased from Fisher Scientific (Hampton, NH).

### Produce and Groundwater Samples

In July 2019, 53 fruit and vegetable samples were collected from five residential gardeners in a PFAS-impacted community near Fayetteville Works. Participants were not compensated for their produce but were informed of the results of produce testing. Data collected in the study were authorized for secondary use in publications by the North Carolina State University institutional review board (IRB number 20444). To protect the privacy of participants, confidentiality of exact locations will be maintained. In brief, Sites A–D are within 2 miles of the Fayetteville Works whereas Site E is within 6 miles. Samples were primarily collected either fresh from residential gardens or from residents’ freezers. The frozen samples were harvested and labeled with the harvest year by residents, allowing us to confirm that the produce was collected from the residential gardens during specific harvest years. Some pickled produce samples (*n* = 6) were collected in sealed jars that had been stored at ambient temperatures. The impact of the pickling process on PFAS content was not investigated in this study because of a lack of control produce. Detailed inventory and descriptions of the produce samples, such as sample weight, are provided in Table S3. Because most produce samples (29 out of 53) were collected frozen and up to 6 years after their original harvest date, the relevance of soil data collected at the time of sampling was deemed minimal. Additionally, our preliminary data indicated high variability in PFAS soil concentrations within the same garden lot, influenced by factors such as sampling location, whether the area was covered by vegetation or was an open area, and likely soil properties. Given the diversity of produce types in this study, which were grown across residential lots ranging in size from 0.5 to ∼5 acres (with an average size of 3.2 acres), it is unlikely that a single or average PFAS concentration value for soil would accurately represent the PFAS levels at each site, particularly over time. Therefore, no efforts were made to link PFAS levels in produce to PFAS concentrations in the garden soils. However, to provide an indication of general environmental levels of PFASs in the vicinity of where the produce was grown, groundwater samples were collected from private wells at each site in July 2019 (Sites A and E) and August 2023 (Sites B, C and D). Samples were collected after flushing the water tap for 3 min. No water treatment devices were installed between the well and the sample tap. Groundwater samples were analyzed using a large-volume injection method without solid-phase extraction (SPE).^21^ According to residents, irrigation in the area primarily relies on natural wet deposition, with negligible use of groundwater for irrigation. It is necessary to point out that this is not a greenhouse study, therefore the impacts of irrigation or precipitation frequency, fertilization, site variation, and other human or environmental variables were not controlled. Water and produce samples were transported back to the laboratory at North Carolina State University in a cooler on the same sampling day and stored at 4 and −20 °C, respectively before analysis.

### Extraction Workflow for Produce Samples

#### Produce Extraction

The extraction workflow consisted of homogenization and extraction using 0.01 M ammonium hydroxide in methanol (basic methanol), following a revised method based on a previous study (Figure S2).^39^ Frozen samples were thawed at room temperature (20 °C) before homogenization. To ensure analysis of representative samples, the entire bag or jar of produce was cut into small pieces using a food chopper in a glass bowl, except for the 2019 blueberry samples collected from Site A. To assess whether dietary exposure through produce can be reduced by washing, a portion of the blueberry samples was washed with water or methanol before homogenization and extraction. For potato and pecan samples, specific masses of deionized water were added to aid in homogenization (Table S3). After thorough mixing, ∼30 g of sample were then homogenized in 50 mL polypropylene centrifuge tubes using a stainless-steel hand-held tissue homogenizer (Omni International, GA). Depending on the dilution ratio in the homogenized samples (Table S3), between 1 to 2 g (±0.01 g) of subsample containing 1 g of the original produce was weighed into 15 mL polypropylene centrifuge tubes, followed by addition of 1 ng of isotopically labeled internal standards (IS). For matrix spike samples, 1 ng of each native PFAS was spiked into randomly selected samples. The spike-recovery was calculated as shown in eq 1. Mass_matrix spike_ and mass_matrix_ represented the masses of PFASs determined in the spiked and non-spiked matrices, respectively.
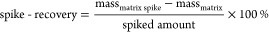
1

The first extraction cycle was performed by adding 4 mL of basic methanol, followed by vortexing, sonicating, and centrifuging. The supernatant was decanted, and the extraction cycle was repeated twice with 2 mL of basic methanol. After three extraction cycles, ∼8.5 mL of supernatant, including native water content of the produce (first supernatant), was collected in clean 15 mL polypropylene tubes, which were stored at −20 °C for 12 h to precipitate any starch present, and then centrifuged at 2800 relative centrifugal force (RCF) for 10 min to separate solids. Around 8 mL of extract (second supernatant) was decanted into clean 250 mL HDPE bottles (Nalgene, Thermo Fisher) for matrix cleanup.

#### Matrix Cleanup and PFAS Enrichment

To clean up produce extracts, the second supernatant was diluted with 150 mL of deionized water to maintain a methanol ratio of 5% or below, which we found was necessary to reduce short-chain PFAS losses during the solid phase extraction (SPE) step. After modifying the original method by decreasing the methanol ratio from 8 to 5%, we found decreasing the methanol content mitigated the loss of short-chain PFASs [e.g., perfluorobutanoic acid (PFBA), PFMOAA, perfluoro-2-methoxypropanoic acid (PMPA)] during SPE and observed a substantial response increase. For example, the peak response of MPFBA (the isotopically labeled standard for quantifying PFBA, PFMOAA and PMPA) increased by a factor of 5.2 when decreasing the methanol ratio from 8 to 5% (Figure S3). Diluted extracts were loaded onto Oasis WAX SPE cartridges (60 mg, 60 μm, Waters Corp., Milford, MA) using an automated SPE system designed for PFAS analysis (Thermo Scientific, Dionex AutoTrace 280 PFAS). SPE cartridges were precleaned with 2 mL of 0.3% NH_4_OH in methanol, then conditioned with 2 mL of methanol and 2 mL of deionized water. After sample loading at a flow rate of 10 mL/min, cartridges were washed with 2 mL of sodium acetate buffer (pH 4.0, 25 mM). Two elution steps (2 mL of methanol followed by 2 mL of 0.3% NH_4_OH in methanol) followed, the two eluents were combined, and samples were evaporated to dryness under gentle ultrahigh purity nitrogen flow at 40 °C for ∼60–90 min. Samples were then reconstituted in 5 mL of 5 mM ammonium acetate in methanol:water (10:90% by volume) for PFAS analysis.

### PFAS Quantification and Quality Control

A liquid chromatography-tandem mass spectrometry (LC-MS/MS) system (1290/6495C Agilent, Santa Clara, CA) equipped with a 4.6 mm × 50 mm LC column (ZORBAX Eclipse Plus C18, 3.5 μm, Agilent) along with a large volume (200 μL) sample injection was used for PFAS analysis.^21^ The column temperature was kept at 50 °C. An additional Agilent ZORBAX Eclipse Plus C18 column was connected before the injector to separate any background PFAS contamination. Detailed instrument parameters are provided in Tables S4 and S5. Calibration standards were reinjected at the end of each batch, with calibration checks conducted after every 20 samples to monitor the stability of the calibration throughout the analysis. The calibration was considered stable if the results fell within a tolerance range of 70–130%.

For quality control, all samples were extracted in triplicate using individual subsamples from the original homogenate and arithmetic means were calculated. Results were consistent among triplicates, with variations within 10% of the mean values, which were adopted for further comparison and analysis in this study. Calibration samples (0.01–5 ng/g) and procedural blanks (homogenized deionized water, n = 3) were spiked with IS and processed following the same workflow. Matrix-matched calibration curves were not used because of the diverse range of produce types included in this study. Only peaks with a signal-to-noise (S/N) ratio >10 were kept for quantification. The method reporting limit (MRL) was determined as the lowest calibration level that could be determined with an accuracy of 70–130% or the average concentration detected in procedural blanks plus 10 times the standard deviation, whichever was greater, as shown in Table S6. Additionally, spike-recoveries of PFASs in 11 matrices (major produce types among all matrices) are shown in Figure S4 and listed in Table S6. For PFASs without corresponding IS, an IS with a similar chromatographic retention time was used to achieve the most accurate recoveries (Table S1).^39^ For PFASs with corresponding IS, recoveries primarily fell in the range of 70–130%, except for 2*H*-perfluoro-2-octenoic acid (6:2 FTUCA) and 2*H*-perfluoro-2-decenoic acid (8:2 FTUCA) in three matrices (corn, peas and pecan), which might be associated with the higher contents of starch or fat in these matrices. For PFASs without corresponding ISs, recoveries ranged from 34 to 502%. For the 13 PFEAs targeted in this study, recoveries generally were between 30 and 200%, with exceptions including PFMOAA in blueberries (233%), perfluoro-3,5,7-trioxaoctanoic acid (PFO3OA) in tomatoes (28%) and Nafion byproduct 2 in blackberries (220%). Detailed spike-recoveries of PFASs in the 11 matrices are provided in Table S6.

### Human Exposure Assessment

To evaluate the relative importance of PFAS exposure from produce to that from drinking water, the water-equivalent daily limit of produce was calculated using eq 2. The average GenX concentration in produce samples was individually calculated for Sites A (*n* = 20), B (*n* = 23) and E (*n* = 7). Calculations were only made for GenX because it was the only regulated PFAS that was frequently detected in the studied produce samples.
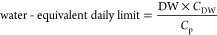
2

DW represents average drinking water ingestion rates, including other liquid formats like soup, which are 1.3 L/day for adults (21 to <50 years) and 0.33 L/day for children (3 to <6 years) as reported in the EPA Exposure Factors Handbook (Chapter 3, 2019).^40^*C*_DW_ represents the maximum allowable GenX concentration in drinking water (10 ng/L),^15^ and *C*_p_ represents the average concentration of GenX in produce harvested from different gardens.

To further assess human exposure to PFASs through consumption of locally grown produce, we calculated the chronic-exposure daily limit for produce using eq 3, based on the chronic reference dose (RfD) for GenX developed by USEPA (0.000003 mg/kg-day).^41^ This limit indicates the maximum amount of produce an individual could consume daily, assuming that all GenX exposure comes exclusively from the produce. BW represents the average body weight of 80 kg for adults or 18.6 kg for children aged 3 to 6 years, as recommended by the EPA Exposure Factors Handbook (Chapter 8, 2011).^42^
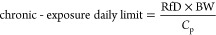
3

#### Statistical Analysis

PFAS concentrations in replicate samples are reported as the arithmetic mean ± one standard deviation. The correlations between different PFAS concentrations in individual produce samples were evaluated using Spearman correlation coefficients (RStudio Software, Version 2023.12.1), and values >0.70 were considered to be highly correlated.

## Results and Discussion

### Site Information and Produce Inventory

The five residential gardens (Sites A, B, C, D, E) were all located in a PFAS-impacted community, where >8000 private wells are contaminated with PFASs, primarily with PFEAs.^43^ As shown in Table 1, PFAS concentrations in private well water at the 4 sites located within 2 miles of the fluorochemical manufacturer were highest at Site A (GenX: 304 ng/L, ΣPFAS: 1209 ng/L) and lowest at Site C (GenX: 3 ng/L, ΣPFAS: 23 ng/L). Sites B and D had similar GenX (24 and 23 ng/L) and ΣPFAS (111 and 159 ng/L) concentrations in the groundwater. Based on NC DEQ and Chemours residential well sampling results (Figure S5), the distribution pattern of PFEAs in the private well water aligns more closely with the dominant wind direction in the region rather than with the distance from the fluorochemical manufacturer.^44^ This observation suggests that air emissions from the fluorochemical manufacturing plant and subsequent dry and wet deposition are the primary contributors to PFEA contamination in groundwater. Additionally, previous studies have detected PFEAs in rainwater and pine needles collected in the area,^45^ providing further evidence of atmospheric deposition. For the well water sampled from Site E (within 6 miles of the plant), GenX was below the MRL (2 ng/L). Site E was intentionally selected as a “lower contamination site” because it is located further from the manufacturer and not in the dominant downwind direction, resulting in overall lower levels of PFEAs in the well water (Table 1). However, multiple legacy PFASs were present (65 ng/L) in the well water from Site E, including PFOS at 33 ng/L (Table S7), which was higher than in well water at the other sites. One or more additional PFAS source(s) may exist near Site E and contribute to the different PFAS signature there. Overall, our groundwater results agreed with NC DEQ well sampling results and previous studies in that low molecular weight PFEAs dominated the PFAS signature.^27^

**Table 1:** Concentrations of Top Five PFEAs and Other PFASs in Private Well Water Samples from the Five Study Sites

		PFAS concentration (ng/L)				
PFAS	MRL (ng/L)	A	B	C	D	E
PFMOAA	5	50	<MRL	<MRL	9	<MRL
PMPA	10	439	45	16	82	26
PEPA	2	159	10	2	16	4
GenX	0.5	304	24	3	23	<MRL
PFO2HxA	2	172	17	<MRL	24	12
other PFASs	/	85	15	2	5	65
sum	/	1209	111	23	159	106

The 53 produce samples obtained at Sites A–E were grouped into water-rich (e.g., blueberries, blackberries, figs; *n* = 39), tree-fruit (apples, pears, peaches; *n* = 8), oil-rich (pecans; *n* = 2), and starch-rich (corn, potatoes, sweet potatoes; *n* = 4) types based on the texture of produce, as shown in Table S3. Among the five sites, Site A (*n* = 20) and Site B (*n* = 23) provided the widest range of sample types and harvesting years from 2013 to 2019. From Site C (*n* = 1), D (*n* = 2), and E (*n* = 7), only water-rich samples harvested in 2019 were collected.

### Overview of PFASs in Homegrown Produce

**Figure 1:**
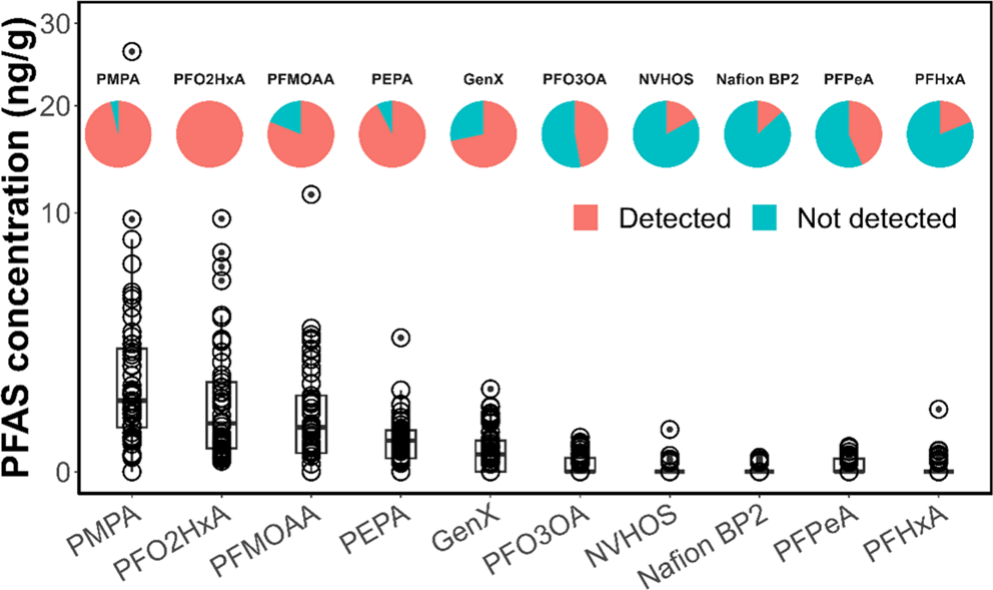
Concentrations and detection frequencies of ten PFASs in fifty-three produce samples collected in an impacted community near a fluorochemical manufacturer in Fayetteville, North Carolina. Only PFASs detected in more than 10% of samples are shown.

Summed PFAS concentrations, dominated by PFEAs, in locally grown produce ranged from 0.026 to 38 ng/g. We detected 10 PFASs, including 8 PFEAs, in at least 10% of the produce samples (Figures 1 and S6). Detection frequencies and statistics of PFAS concentration are summarized in Table S8. Five low molecular weight PFEAs [PMPA, PFO2HxA, perfluoro-2-ethoxypropanoic acid (PEPA), PFMOAA, GenX] were detected in over 70% of the produce samples. We found a strong correlation between the five PFEAs that were frequently detected, suggesting the five PFEAs tended to coexist in the produce (Figure S7). In particular, PFO2HxA was detected in all samples, and PMPA was detected in 96% (51:53). Three low molecular weight PFEAs—PMPA, PFO2HxA, and PFMOAA (structures shown in Figure S1)—were detected with mean concentrations of 2.1, 1.2, and 0.9 ng/g, respectively (Table S8). Despite PFMOAA having substantially lower concentrations in groundwater samples (Table 1), its mean concentration in produce samples was 6 times that of GenX. This result suggests that short-chain PFEAs, such as PFMOAA, are readily taken up by plants, similar to short-chain PFCAs and PFSAs.^36,46^

Among traditionally studied PFCAs and PFSAs, perfluoropentanoic acid (PFPeA) and perfluorohexanoic acid (PFHxA) were the only two detected in >10% of the samples, with mean concentrations of 0.016 and 0.018 ng/g, respectively. These concentrations are 2 orders of magnitude lower than those of the dominant PFEAs (Table S8). Suspected PFBA peaks were observed in some matrices with retention time shifts greater than 0.1 min compared to the MPFBA peak. Additionally, the same peak appeared with a shoulder in the matrix-spike sample (Figure S7), suggesting it may be an interference peak with the same *m*/*z*, overlapping with the true PFBA peak. Because PFBA only has one mass transition, a qualifier ion to confirm the peak was not available; therefore, we excluded PFBA data from further analysis.

### Effect of Produce Type

**Figure 2:**
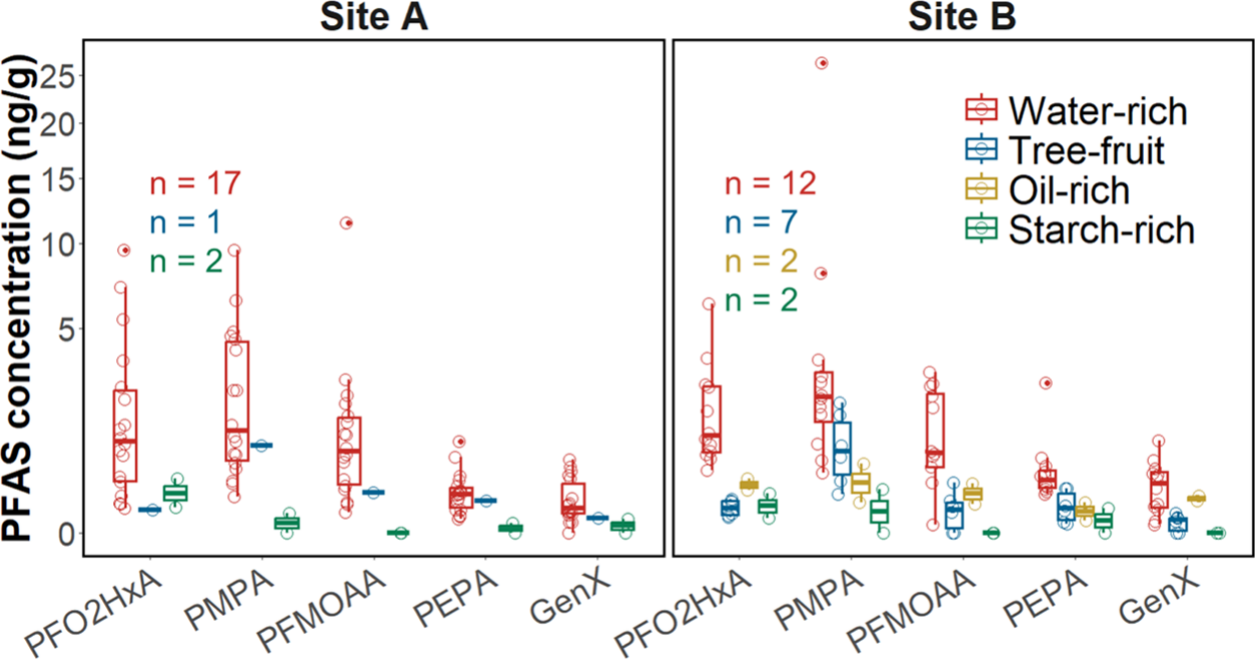
Effect of produce type on PFAS concentrations in produce. The sample size for each box plot was labeled using colors that match the corresponding scatter points.

PFAS concentrations in produce varied substantially across different types of samples. To rule out the effect of sampling site, samples from Sites A (*n* = 20) and B (*n* = 23) were grouped into water-rich, tree-fruit, oil-rich, and starch-rich samples for comparison (Table S3). The effect of produce type was not investigated for samples from Sites C (*n* = 1), D (*n* = 2), and E (*n* = 7) because a limited number of samples from only one sample type (water-rich produce) was collected from these sites. The relationship between produce type and PFAS concentrations in produce is shown in Figure 2. On average, water-rich samples such as blueberries, blackberries and squash had the highest concentrations of PFASs, whereas starch-rich samples such as corn and potatoes had lower levels. This finding agrees with previous studies, which found PFASs are more enriched in water-rich produce such as strawberries, tomatoes, and lettuce as compared to starch-rich produce, such as corn kernels.^36,46^ For tree-fruits such as apples and peaches, which are also water-rich, PFAS levels were substantially lower than in other water-rich produce samples, likely due to the long transport route from the root to fruits.^47^ Figs sampled from Site B were an exception with a summed PFAS concentration as high as 38 ng/g (outlier in Figure 2b). Because fig trees in North Carolina often grow as large shrubs, rather than as trees with a single trunk,^48^ we grouped figs into the water-rich group instead of tree-fruits in this study. For samples low in water, but high in oil or starch, we detected PFASs at lower concentrations, suggesting water content might be a critical factor in considering PFAS uptake into plant compartments. The low PFEA uptake by corn observed in our study is consistent with a previous study that found lower uptake of PFCAs and PFSAs by corn compared to tomato and lettuce, despite similar soil concentrations.^46^

### Temporal and Spatial Trends

**Figure 3:**
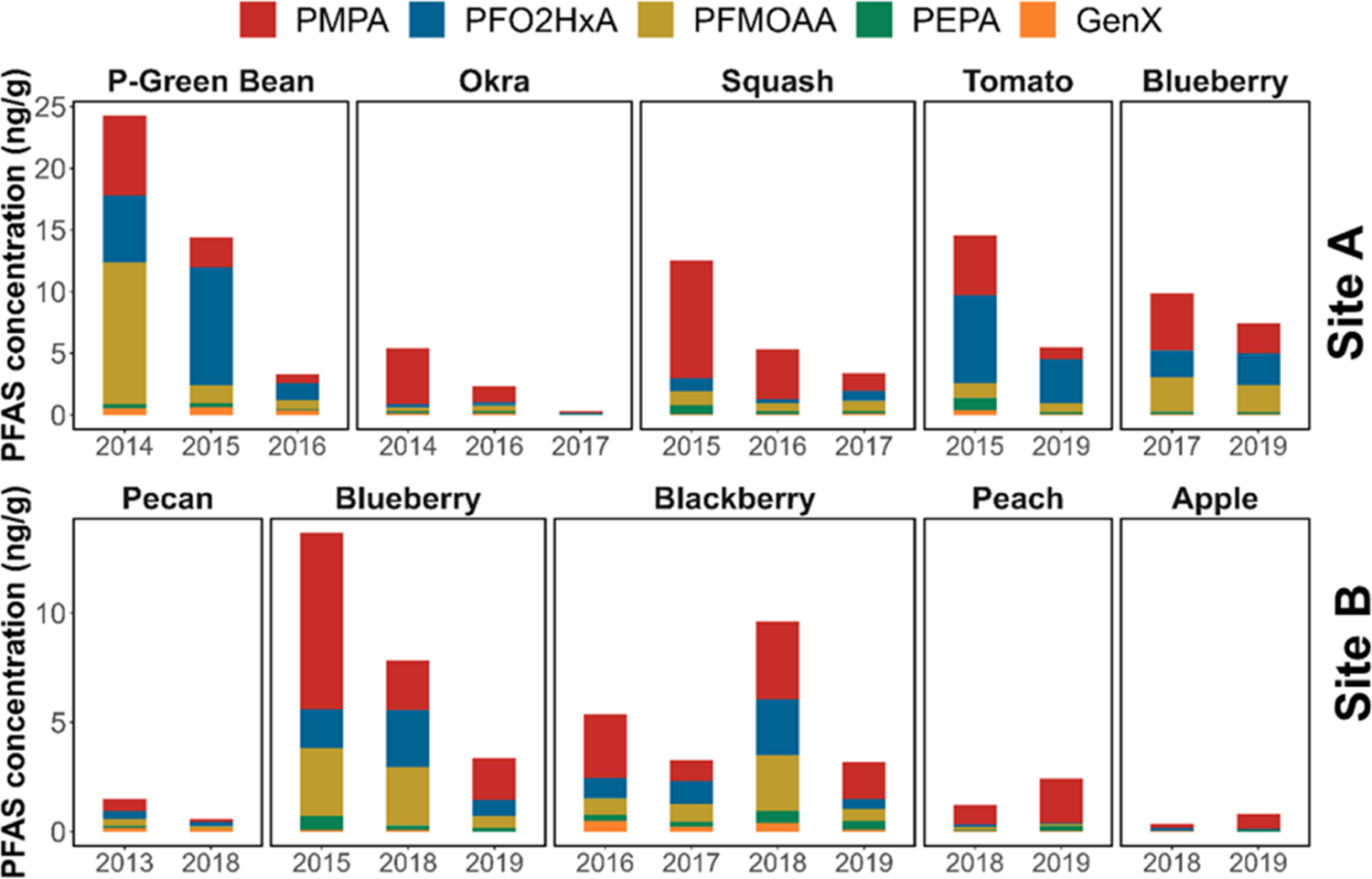
Temporal PFAS concentration trends in different produce types at two sampling sites.

Samples from multiple harvesting years were obtained at Sites A and B. The temporal trends of PFAS concentrations in a given type of produce are shown in Figure 3. At Site A, PFEA concentrations in five produce types (pickled green beans, okra, squash, tomato and blueberry) decreased substantially from 2014 to 2019, whereas the trend among produce samples collected from Site B (pecan, blueberry, blackberry, peach and apple) was less clear. As a function of collection year at Site B, we observed decreasing PFEA concentrations in pecan (2013, 2018) and blueberry (2015, 2018, 2019) samples but increasing PFAS concentrations in blackberry (from 2017 to 2018) as well as apple and peach samples (from 2018 to 2019).

It is unclear what might have led to the overall declining trend of PFEA concentrations in the produce. Considering that gardens in the area are primarily rainfed, we suspect that declining air emissions from the fluorochemical manufacturer might have played a role in the observed PFAS levels. In 2013, the fluorochemical manufacturer implemented an abatement technology, which may have reduced PFAS emissions to the atmosphere and hence subsequent PFAS deposition from air to the land surface in the vicinity of the PFAS manufacturing facility.^49^ According to air quality sampling conducted by the NC DEQ, the atmospheric deposition of GenX has generally decreased from 2018 to 2022, following the installation of additional air pollution control devices at Fayetteville Works.^50^ However, due to the absence of atmospheric deposition data prior to 2018, it is difficult to evaluate how environmental exposure to PFASs changed over the harvest years (2013–2019). Reduced atmospheric deposition may have contributed to decreasing PFEA concentrations near the land surface (e.g., in root zone soil and pore water). Compared to groundwater, PFAS concentrations in root zone pore water may change more quickly as a result of shorter transport distances. Thus, PFAS uptake by plants may respond more quickly to lower air emissions than PFAS concentrations in private well water. Overall, analysis of additional samples is needed to more clearly establish whether PFAS levels in fruits and vegetables grown near the fluorochemical manufacturer are decreasing as a result of interventions that have been implemented to reduce air emissions.

**Figure 4:**
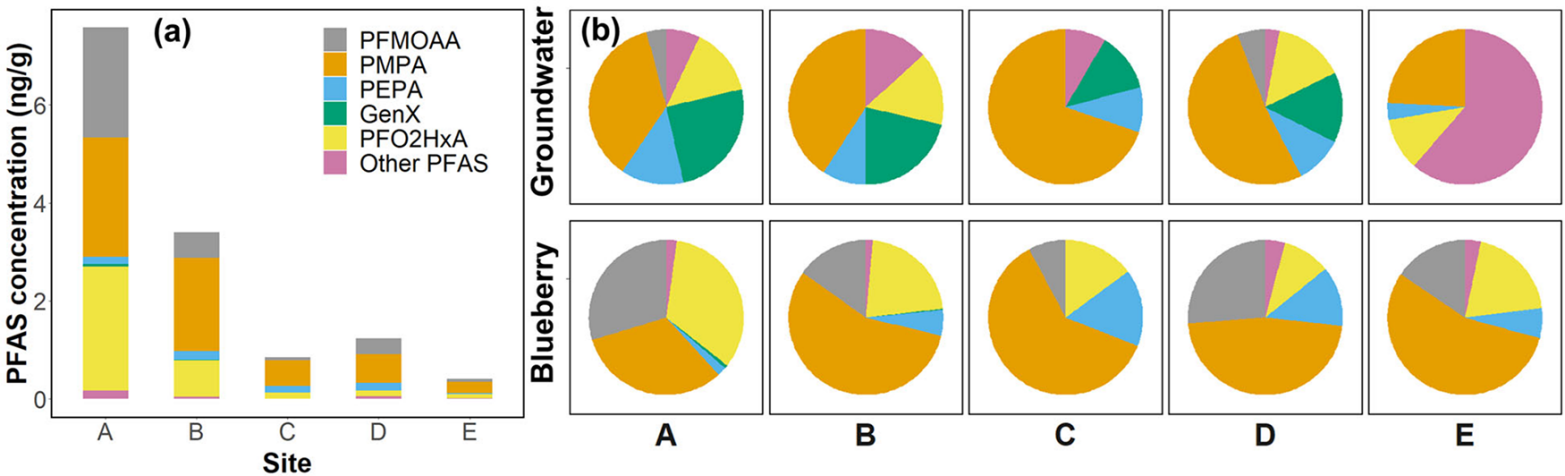
(a) PFAS concentrations in blueberry samples harvested from different sites in 2019 and (b) comparison of PFAS signatures in groundwater and blueberry samples collected from different sites.

With respect to spatial distribution, like the groundwater data, the distance between a site and the fluorochemical plant did not explain the differences in PFAS concentrations in the produce, in contrast to a previous study in The Netherlands.^51^ Sites A–D are all within a radius of 2 miles of Fayetteville Works, but PFAS concentrations in produce samples varied substantially across sites, similar to what was observed in the groundwater results. To rule out the effect of produce type and sampling time, PFAS concentrations in blueberry samples harvested in 2019 from different sites were compared in Figure 4a. The summed concentration of PFASs in blueberries ranged from 0.4 to 7.6 ng/g, which partially agreed with water sampling results (Table 1), with Site A blueberries exhibiting the highest PFEA concentrations in both the groundwater and blueberries. However, PFAS concentrations in Site B blueberries were much higher than those in Site D blueberries despite the similar PFAS concentrations in Site B and D groundwater.

Overall, PFAS signatures in blueberries differed from those in groundwater (Figure 4b). For example, concentrations of PFMOAA and PFO2HxA in the groundwater at Site C were both below the MRL, whereas PFMOAA and PFO2HxA accounted for about 25% of the summed PFAS concentration in the blueberries. Conversely, at Site E, PFASs other than the five PFEAs—primarily PFOS and legacy PFASs—comprised over 60% of the summed PFAS concentration in the groundwater yet accounted for less than 5% of the PFAS concentration in the blueberries. Although higher PFAS concentrations in groundwater generally corresponded to higher concentrations in blueberries (Figure S9), no clear correlation was observed, particularly for PMPA and PEPA. These discrepancies suggest that the groundwater concentration alone is not an effective predictor of PFEA concentrations in produce. PFAS concentrations between produce samples and groundwater samples may differ because individual PFASs are taken up by and transported differently within different plants.^46,52^ Also, PFAS concentrations in the groundwater may differ from those in pore water in the root zone near the land surface (not measured, historical samples not available); PFASs in pore water likely represent the dominant fraction bioavailable for plant uptake. For instance, a previous study found no apparent relationship between pharmaceutical concentrations in the soil and their accumulation in radishes, but a strong positive correlation was observed between the accumulation of pharmaceuticals in radishes and the corresponding pharmaceutical concentrations in soil pore water.^53^ This finding suggests that the bioavailability of contaminants to plants might be better represented by soil pore water concentrations rather than groundwater concentrations. Additionally, the differential retention of PFASs by soil could alter the PFAS profile between soil pore water and groundwater, i.e., shorter-chain PFASs tend to have lower retardation factors compared to longer-chain PFASs.^54^ Variations in soil physiochemical characteristics, such as organic matter content, could also affect PFAS bioavailability.^55^ However, soil composition was not analyzed in this study, and we cannot evaluate the specific impact of this factor. Furthermore, other uncontrolled biological and environmental factors, such as wind direction, landscape position, irrigation frequency, age, variety, and physiology of the blueberry bushes may contribute to such discrepancy. Overall, our results indicate that groundwater is not an effective predictor of PFAS levels and signatures in produce; nonetheless, shallow groundwater can be a useful first-level indicator whether PFAS contamination of produce may be a concern at a particular location.

### Human Exposure Assessment

**Figure 5:**
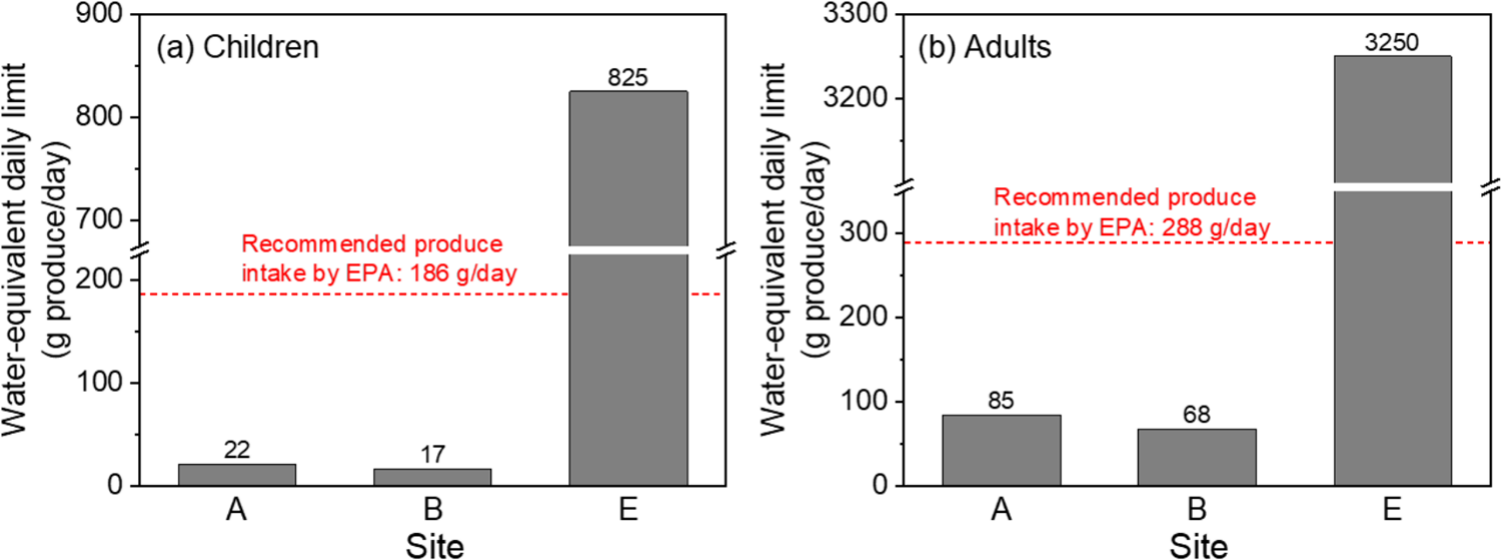
Equivalent amount of produce that would cause the same level of PFEA exposure as consuming drinking water containing 10 ng/L GenX for (a) children and (b) adults. The dashed lines indicate the recommended produce intake for children (3 to <6 years) and adults (21 to <50 years) according to the EPA Exposure Factors Handbook.

Elevated concentrations of PFEAs in private garden produce near the fluorochemical manufacturer suggest dietary exposure can contribute substantially to human exposure to PFASs in impacted communities. To date, regulations on limiting PFAS concentrations in food are lacking. Because GenX is the only regulated PFAS with elevated and widespread detection (>70%) in our samples, we calculated the average GenX concentration in produce samples collected from Site A, B and E as 0.15, 0.19, and 0.004 ng/g, respectively. Sites C (*n* = 1) and D (*n* = 2) were not included because of their small sample sizes. To compare the relative importance of dietary exposure via produce and drinking water exposure, we calculated the water-equivalent daily limits for consuming produce harvested from Sites A, B and E using eq 2. We assumed that the drinking water at these sites contained 10 ng/L of GenX, the maximum allowable concentration regulated by the U.S. EPA.^15^ We then calculated the equivalent amount of produce that would cause the same level of PFEA exposure as consuming drinking water containing 10 ng/L GenX. As shown in Figure 5, for the less contaminated produce from Site E, GenX exposure through drinking water is equivalent to consuming 3250 and 825 g/day of produce for adults aged 21 to 50 years and children aged 3 to 6 years, respectively, which greatly exceeds typical produce intake rates. However, for produce harvested at Sites A and B, the daily GenX exposure through drinking water is equivalent to children consuming 22 and 17 g/day of produce, or adults consuming 85 and 68 g/day of produce, respectively. These amounts are substantially lower than EPA recommended intake values for fruits and vegetables, approximately 9 times lower for children and 4 times lower for adults. According to the EPA Exposure Factors Handbook, the recommended intake is 186 g/day for children aged 3–6 years (with a body weight of 18.4 kg) and 288 g/day for adults aged 21–50 years (with a body weight of 80 kg).^37,42^ These findings highlight that consumption of contaminated produce can be an important route of PFAS exposure in impacted communities, especially for children.

To assess the long-term risk of consuming contaminated produce in impacted communities, we calculated a chronic-exposure daily limit—the maximum amount of produce that an individual could consume daily, assuming all GenX exposure comes from the produce—based on the chronic chemical reference dose (RfD) of GenX (0.000003 mg/kg-day), as shown in eq 3. The chronic-exposure daily limit for Site A and B produce were 367 and 289 g/day, respectively, for children aged 3 to 6 years (Table 2). While these amounts are higher than the recommended value (186 g/day), chronic effects may still occur by regularly consuming contaminated homegrown fruits and vegetables. Meanwhile, the adult chronic-exposure daily limits for produce from Sites A and B were 1579 and 1244 g/day, respectively. These limits are substantially higher than the recommended value in the EPA Exposure Factors Handbook (3.6 g/kg body weight-day for the sum of fruits and vegetables or ∼288 g/day for a typical 80 kg adult).^37^ However, the chronic-exposure daily limit was calculated based on GenX only, which represented a small fraction of total quantified PFASs in the studied produce. We may therefore underestimate risk because we are not considering potentially additive effects resulting from PFAS mixtures,^56^ especially for PFEAs that were detected at concentrations higher than GenX and for which RfD values are lacking.

**Table 2:** Chronic-Exposure Daily Limit for Produce Harvested from Site A, B and E^a^

		chronic-exposure daily limit (g produce/day)	
site	average GenX concentration in produce (ng/g)	children	adults
A	0.152	367	1579
B	0.193	289	1244
E	0.004	13,950	60,000

a Note: The recommended values for intake of fruits and vegetables given by the EPA Exposure Factors Handbook are 186 and 288 g/day of produce for children (3 to <6 years) and adults (21 to <50 years), respectively.^37^

Finally, our results suggest washing is not effective in reducing PFAS concentrations in produce. The majority (>85%) of PFASs in the blueberry samples collected from Site A in 2019 were preserved after intensive washing with water or methanol, as shown in Figure S8, suggesting PFASs had primarily accumulated through internal plant uptake, instead of through atmospheric deposition onto the external surface of produce. For all other samples, we prepared and extracted the produce samples as received, without washing. Washing might have occurred during the original collection and storage process performed by the residents, but our results suggest that washing would not have substantially altered PFAS concentrations. Overall, our results indicate that PFAS exposure through homegrown produce may be substantial for people living in PFAS-impacted communities, particularly for children. However, a more comprehensive exposure assessment is needed for a complete evaluation.

## Implications

Drinking water has been identified as the major PFAS exposure pathway for people living downstream of a fluorochemical manufacturer in North Carolina. However, for people living near the manufacturer and at the frontline of PFAS contamination, exposure through locally grown produce can be important, especially for children. Among different types of plants, we observed varying levels of PFAS enrichment in the edible parts. Some plants, such as those yielding oil-rich and starch-rich produce tend to exhibit lower PFEA concentrations in their edible parts than water-rich produce. This information can support risk communication and the development of guidelines for home produce production and consumption in contaminated areas.

## Supporting Information Available

Details regarding the targeted PFAS list, sample preparation method, LC-MS method, quality control and quality assurance (QA/QC) data, PFAS concentrations in produce and groundwater samples, structures of the dominant PFASs in produce and groundwater, statistical analysis of the data (PDF)
