# Supplementary material for: Residential Garden Produce Harvested Near a Fluorochemical Manufacturer in North Carolina Can Be An Important Fluoroether Exposure Pathway
Source: J Agric Food Chem. 2024 Nov 20;72(48):26874–83. doi: 10.1021/acs.jafc.4c06177 (PMC11622232; doi:10.1021/acs.jafc.4c06177)
Supplement: Supplementary file 1 — jf4c06177_si_001.pdf [file jf4c06177_si_001.pdf]

Supporting information for:

Residential garden produce harvested near a fluorochemical manufacturer in North Carolina can be an important fluoroether exposure pathway

Pingping Meng,[*,1,2,3] *Nadia Sheppard,*[2] *Sarangi Joseph,*[2] *Owen W. Duckworth,*[3,4] *Christopher P. Higgins*[5] *and Detlef R.U. Knappe*[2,3]

[1] Department of Chemistry, East Carolina University, Greenville, NC 27858, USA

[2] Department of Civil, Construction, and Environmental Engineering, North Carolina State University, Raleigh, NC 27695, USA

[3] Center for Human Health and the Environment, North Carolina State University, Raleigh, NC 27695, USA

[4] Department of Crop and Soil Sciences, North Carolina State University, Raleigh, NC 27695, USA

[5] Department of Civil and Environmental Engineering, Colorado School of Mines, Golden, CO 80401 USA

*Email: mengp22@ecu.edu

Table S1. Per- and polyfluoroalkyl substances (PFASs) targeted in this study

Analyte	Formula	CAS # (hyperlinked to US EPA Chemicals Dashboard)	Source[a]	Mass Labelled IS
**Perfluoroalkyl carboxylic acids (PFCAs)**				
Perfluorobutanoic acid (PFBA)	$C_4HF_7O_2$	375-22-4	1	MPFBA
Perfluoropentanoic acid (PFPeA)	$C_5HF_9O_2$	2706-90-3	1	M5PFPeA
Perfluorohexanoic acid (PFHxA)	$C_6HF_{11}O_2$	307-24-4	1	M5PFHxA
Perfluoroheptanoic acid (PFHpA)	$C_7HF_{13}O_2$	375-85-9	1	M4PFHpA
Perfluorooctanoic acid (PFOA)	$C_8HF_{15}O_2$	335-67-1	1	M8PFOA
Perfluorononanoic acid (PFNA)	$C_9HF_{17}O_2$	375-95-1	1	M9PFNA
Perfluorodecanoic acid (PFDA)	$C_{10}HF_{19}O_2$	335-76-2	1	M6PFDA
Perfluoroundecanoic acid (PFUnDA)	$C_{11}HF_{21}O_2$	2058-94-8	1	M7PFUndA
Perfluorododecanoic acid (PFDoA)	$C_{12}HF_{23}O_2$	307-55-1	1	M2 PFDoA
Perfluorotridecanoic acid (PFTrDA)	$C_{13}HF_{25}O_2$	72629-94-8	1	M2 PFTeDA
Perfluorotetradecanoic acid (PFTeDA)	$C_{14}HF_{27}O_2$	376-06-7	1	M2 PFTeDA
**Perfluoroalkyl sulfonic acids (PFSAs)**				
Perfluorobutanesulfonic acid (PFBS)	$C_4HF_9O_3S$	375-73-5	1	M3PFBS
Perfluoropentanesulfonic acid (PFPeS)	$C_5HF_{11}O_3S$	2706-91-4	1	M3PFHxS
Perfluorohexanesulfonic acid (PFHxS)	$C_6HF_{13}O_3S$	355-46-4	1	M3PFHxS
Perfluoroheptanesulfonic acid (PFHpS)	$C_7HF_{15}O_3S$	375-92-8	1	M8PFOS
Perfluorooctanesulfonic acid (PFOS)	$C_8HF_{17}O_3S$	1763-23-1	1	M8PFOS
Perfluorononanesulfonic acid (PFNS)	$C_9HF_{19}O_3S$	68259-12-1	1	M2 8:2 FTS
Perfluorodecanesulfonic acid (PFDS)	$C_{10}HF_{21}O_3S$	2806-15-7	1	d5-N-EtFOSAA
**Per- and polyfluoroalkyl ether carboxylic acids (PFECAs)**				
Perfluoro-2-methoxyacetic acid (PFMOAA)	$C_3HF_5O_3$	674-13-5	2	MPFBA
Perfluoro-2-methoxypropanoic acid (PMPA)	$C_4HF_7O_3$	13140-29-9	3	MPFBA
Perfluoro-2-ethoxypropanoic acid (PEPA)	$C_5HF_9O_3$	267239-61-2	3	M5PFPeA

Perfluoro-2-propoxypropanoic acid (PFPrOPrA) = Hexafluoropropylene oxide-dimer acid (HFPO-DA) = parent acid of "GenX"	$C_6HF_{11}O_3$	13252-13-6	1	M3HFPO-DA
Perfluoro-3,5-dioxahexanoic acid (PFO2HxA)	$C_4HF_7O_4$	39492-88-1	3	M5PFPeA
Perfluoro-3,5,7-trioxaoctanoic acid (PFO3OA)	$C_5HF_9O_5$	39492-89-2	3	M5PFHxA
Perfluoro-3,5,7,9-butaoxadecanoic (PFO4DA)	$C_6HF_{11}O_6$	39492-90-5	3	M4PFHpA
Perfluoro-3,5,7,9,11-pentaoxadodecanoic acid (PFO5DoA)	$C_7HF_{13}O_7$	39492-91-6	3	M9PFNA
2,2,3,3-Tetrafluoro-3-{[1,1,1,2,3,3-hexafluoro-3-(1,2,2,2-tetrafluoroethoxy)propan-2-yl]oxy}propanoic acid (HydroEVE)	$C_8H_2F_{14}O_4$	773804-62-9	3	M4PFHpA
Sodium dodecafluoro-3H-4,8-dioxanonanoate (sodium salt of "ADONA")	$C_7HF_{12}NaO_4$	958445-44-8	1	M4PFHpA
**Per- and polyfluoroalkyl ether sulfonic acids (PFESAs)**				
Perfluoro-2-{[perfluoro-3-(perfluoroethoxy)-2-propanyl]oxy}ethanesulfonic acid (Nafion by-product 2)	$C_7H_2F_{14}SO_5$	749836-20-2	3	M3PFHxS
1,1,2,2-tetrafluoro-2-(1,2,2,2-tetrafluoro-ethoxy)ethane sulfonic acid (NVHOS)	$C_4H_2F_8O_4S$	1132933-86-8	3	M3PFBS
9-chlorohexadecafluoro-3-oxanonane-1-sulfonate (9Cl-PF3ONS, major component of F-53B)	$C_8HF_{16}O_4SCl$	73606-19-6	1	M6PFDA
**Perfluoroalkane sulfonamido acetic acids (FASAAs)**				
N-methyl perfluorooctanesulfonamidoacetic acid (N-MeFOSAA)	$C_{11}H_6F_{17}NO_4S$	2355-31-9	1	d3-N-MeFOSAA
N-ethyl perfluorooctanesulfonamidoacetic acid (N-EtFOSAA)	$C_{12}H_8F_{17}NO_4S$	2991-50-6	1	d5-N-EtFOSAA
**Perfluoroalkane sulfonamides (FASAs)**				
Perfluorobutane sulfonamide (PFBSA)	$C_4H_2F_9NO_2S$	30334-69-1	1	M3PFHxS
Perfluorohexane sulfonamide (PFHxSA)	$C_6H_2F_{13}NO_2S$	41997-13-1	1	M8PFOS

Perfluorooctane sulfonamide (PFOSA)	$C_8H_2F_{17}NO_2S$	754-91-6	1	M8PFOSA
**Fluorotelomer sulfonic acids (FTSs)**				
4:2 Fluorotelomer sulfonic acid (4:2 FTS)	$C_6H_5F_9O3S$	757124-72-4	1	M2 4:2 FTS
6:2 Fluorotelomer sulfonic acid (6:2 FTS)	$C_8H_5F_{13}O3S$	27619-97-2	1	M2 6:2 FTS
8:2 Fluorotelomer sulfonic acid (8:2 FTS)	$C_{10}H_5F_{17}O3S$	39108-34-4	1	M2 8:2 FTS
**Fluorotelomer carboxylic acids (FTCAs)**				
2H,2H-Perfluorooctanoic acid (6:2 FTCA)	$C_8H_3F_{13}O_2$	53826-12-3	1	M2 6:2 FTCA
2H,2H-Perfluorodecanoic acid (8:2 FTCA)	$C_{10}H_3F_{17}O_2$	27854-31-5	1	M2 8:2 FTCA
2H-Perfluoro-2-Octenoic acid (6:2 FTUCA)	$C_8H_2F_{12}O_2$	70887-88-6	1	M2 6:2 FTUCA
2H-Perfluoro-2-Decenoic acid (8:2 FTUCA)	$C_{10}H_2F_{16}O_2$	70887-84-2	1	M2 8:2 FTUCA

[a] Standard source: 1 Wellington Laboratories (Guelph, ON, Canada), 2 Fluoryx Labs (Carson City, NV), 3 The Chemours Company (Wilmington, DE).

Table S2. Mass-labelled analytes used in this study

Abbreviation	Mass labelled analyte	Mass-labelled atom
	**Mass-labelled perfluoroalkyl carboxylic acids**	
MPFBA	Perfluoro-n-[$^{13}C_4$]butanoic acid	$^{13}C_4$
M5PFPeA	Perfluoro-n-[$^{13}C_5$]pentanoic acid	$^{13}C_5$
M5PFHxA	Perfluoro-n-[1,2,3,4,6-$^{13}C_5$]hexanoic acid	1,2,3,4,6-$^{13}C_5$
M4PFHpA	Perfluoro-n-[1,2,3,4-$^{13}C_4$]heptanoic acid	1,2,3,4-$^{13}C_4$
M8PFOA	Perfluoro-n-[$^{13}C_8$]octanoic acid	$^{13}C_8$
M9PFNA	Perfluoro-n-[$^{13}C_9$]nonanoic acid	$^{13}C_9$
M6PFDA	Perfluoro-n-[1,2,3,4,5,6-$^{13}C_6$]decanoic acid	1,2,3,4,5,6-$^{13}C_6$
M7PFUndA	Perfluoro-n-[1,2,3,4,5,6,7-$^{13}C_7$]undecanoic acid	1,2,3,4,5,6,7-$^{13}C_7$
M2 PFDoA	Perfluoro-n-[1,2-$^{13}C_2$]dodecanoic acid	1,2-$^{13}C_2$
M2 PFTeDA	Perfluoro-n-[1,2-$^{13}C_2$]tetradecanoic acid	1,2-$^{13}C_2$
	**Mass-labelled perfluoroalkyl sulfonates**	
M3PFBS	Sodium perfluoro-1-[2,3,4-$^{13}C_3$]butanesulfonate	2,3,4-$^{13}C_3$
M3PFHxS	Sodium perfluoro-1-[1,2,3-$^{13}C_3$]hexanesulfonate	1,2,3-$^{13}C_3$
M8PFOS	Sodium perfluoro-1-[$^{13}C_8$]octanesulfonate	$^{13}C_8$
	**Mass-labelled per- and polyfluoroalkyl ether carboxylic acids (PFECAs)**	
M3HFPO-DA	2,3,3,3-Tetrafluoro-2-(1,1,2,2,3,3,3-heptafluoropropoxy)-$^{13}C_3$-propanoic acid	$^{13}C_3$
	**Mass-labelled fluorotelomer carboxylic acids**	
M2 6:2 FTCA	2-Perfluorohexyl-[1,2-$^{13}C_2$]-ethanoic acid	1,2-$^{13}C_2$
M2 8:2 FTCA	2-Perfluorooctyl-[1,2-$^{13}C_2$]-ethanoic acid	1,2-$^{13}C_2$
M2 6:2 FTUCA	2H-Perfluoro-[1,2-$^{13}C_2$]-2-octenoic acid	1,2-$^{13}C_2$
M2 8:2 FTUCA	2H-Perfluoro-[1,2-$^{13}C_2$]-2-decenoic acid	1,2-$^{13}C_2$
	**Mass-labelled fluorotelomer sulfonic acids**	
M2 4:2 FTS	Sodium 1H,1H,2H,2H-perfluoro-1-[1,2-$^{13}C_2$]-hexane sulfonate	1,2-$^{13}C_2$
M2 6:2 FTS	Sodium 1H,1H,2H,2H-perfluoro-1-[1,2-$^{13}C_2$]-octane sulfonate	1,2-$^{13}C_2$

M2 8:2 FTS	Sodium 1H,1H,2H,2H-perfluoro-1-[1,2-$^{13}C_2$]-decane sulfonate	1,2-$^{13}C_2$
	**Mass-labelled perfluoroalkyl sulfonamido acetic acid**	
d3-N-MeFOSAA	N-methyl-d3-perfluoro-1-octanesulfonamidoacetic acid	98% $^2H_3$
d5-N-EtFOSAA	N-ethyl-d5-perfluoro-1-octanesulfonamidoacetic acid	98% $^2H_5$
	**Mass-labelled perfluoroalkyl sulfonamide**	
M8PFOSA	Perfluoro-1-[^{13}C8]octanesulfonamide	^{13}C8

Table S3. Produce inventory.

Produce	Site	Harvest year	Sample weight (g)	Water added (g)	Extraction size (g)	Produce type
Pickled green beans	A	2014	856	0	1	Water-rich
Pickled okra	A	2014	663	0	1	Water-rich
Pickled green beans	A	2015	824	0	1	Water-rich
Pickles	A	2015	562	0	1	Water-rich
Squash	A	2015	239	0	1	Water-rich
Tomato sauce	A	2015	961	0	1	Water-rich
Okra	A	2016	301	0	1	Water-rich
Pickled beans	A	2016	872	0	1	Water-rich
Pickled green beans	A	2016	864	0	1	Water-rich
Squash	A	2016	336	0	1	Water-rich
Blueberry	A	2017	351	0	1	Water-rich
Corn	A	2017	150	0	1	Starch-rich
Okra	A	2017	341	0	1	Water-rich
Potato	A	2017	315	157.5	1.5	Starch-rich
Squash	A	2017	460	0	1	Water-rich
Blueberry-ripe	A	2019	130	0	1	Water-rich
Blueberry-unripe	A	2019	10	0	1	Water-rich
Grape	A	2019	232	0	1	Water-rich
Peach	A	2019	386	0	1	Tree-fruit
Tomato	A	2019	320	0	1	Water-rich
Pecan	B	2013	169	169	2	Oil-rich
Tomato	B	2013	667	0	1	Water-rich
Blueberry	B	2015	327	0	1	Water-rich
Peas	B	2015	217	0	1	Water-rich
Blackberry	B	2016	342	0	1	Water-rich
Blackberry	B	2017	385	0	1	Water-rich
Turnip	B	2017	98	0	1	Water-rich
Apple	B	2018	242	0	1	Tree-fruit

Blackberry	B	2018	244	0	1	Water-rich
Blueberry	B	2018	328	0	1	Water-rich
Corn	B	2018	275	0	1	Starch-rich
Peach	B	2018	329	0	1	Tree-fruit
Pecan	B	2018	182	182	2	Oil-rich
Sweet potato	B	2018	235	0	1	Starch-rich
Apple-ripe	B	2019	347	0	1	Tree-fruit
Apple-unripe	B	2019	342	0	1	Tree-fruit
Blackberry	B	2019	339	0	1	Water-rich
Blueberry	B	2019	216	0	1	Water-rich
Fig	B	2019	82	0	1	Water-rich
Grape	B	2019	115	0	1	Water-rich
Peach-ripe	B	2019	367	0	1	Tree-fruit
Peach-unripe	B	2019	330	0	1	Tree-fruit
Pear	B	2019	341	0	1	Tree-fruit
Blueberry	C	2019	343	0	1	Water-rich
Blueberry	D	2019	328	0	1	Water-rich
Cucumber	D	2019	99	0	1	Water-rich
Blueberry	E	2019	43	0	1	Water-rich
Cantaloupe	E	2019	293	0	1	Water-rich
Cherry tomato	E	2019	66	0	1	Water-rich
Fig	E	2019	145	0	1	Water-rich
Okra	E	2019	59	0	1	Water-rich
Tomato	E	2019	254	0	1	Water-rich
Watermelon	E	2019	150	0	1	Water-rich

Table S4. LC/MS instrumental parameters

<table>
<tr><th></th><th>Parameters</th><th colspan="4">Values</th></tr>
<tr><td rowspan="6">Ion Source</td><td>Gas temp (°C)</td><td colspan="4">100</td></tr>
<tr><td>Gas flow (L/min)</td><td colspan="4">15</td></tr>
<tr><td>Nebulizer (psi)</td><td colspan="4">15</td></tr>
<tr><td>Sheath gas temp (°C)</td><td colspan="4">250 (Low Temp); 400 (High Temp)</td></tr>
<tr><td>Capillary (V)</td><td colspan="4">3000(+), 2000(-)</td></tr>
<tr><td>Delta EMV</td><td colspan="4">200</td></tr>
<tr><td>Multisampler</td><td>Injection volume (µL)</td><td colspan="4">200</td></tr>
<tr><td rowspan="9">Binary pump</td><td>Flow rate (mL/min)</td><td colspan="4">0.7</td></tr>
<tr><td rowspan="2">Mobile phase</td><td colspan="4">A: 5 mM ammonium acetate in water</td></tr>
<tr><td colspan="4">B: 5 mM ammonium acetate in water/methanol 95/5</td></tr>
<tr><td rowspan="6">Mobile phase gradient</td><td></td><td>Time</td><td>A (%)</td><td>B(%)</td></tr>
<tr><td>1</td><td>0.0</td><td>95</td><td>5</td></tr>
<tr><td>2</td><td>18.0</td><td>0</td><td>100</td></tr>
<tr><td>3</td><td>22.0</td><td>0</td><td>100</td></tr>
<tr><td>4</td><td>22.1</td><td>95</td><td>5</td></tr>
<tr><td>5</td><td>28</td><td>95</td><td>5</td></tr>
</table>

Table S5. PFAS analyte MS/MS parameters

Compound	Source temperature [a]	Mass transition	Dwell time (ms)	Fragmentation energy (V)	Collision energy (V)	Retention Time (min)
		**Perfluoroalkyl carboxylic acids (PFCAs)**				
PFBA	LT	213>169	43	166	6	5.834
PFPeA	LT	262.97>219.04	43	166	8	10.966
PFHxA	LT	312.97>269*	73	166	10	13.483
		312.97>118.9	73	166	22	13.483
PFHpA	LT	362.97>318.8*	17	166	10	15.006
		362.97>168.9	17	166	18	15.006
PFOA	LT	412.96>368.8*	17	166	10	16.127
		412.96>168.9	17	166	18	16.127
PFNA	LT	462.96>419*	19	166	10	17.033
		462.96>218.9	19	166	14	17.033
PFDA	LT	512.96>469*	19	166	10	17.796
		512.96>269	19	166	14	17.796
PFUnDA	LT	562.95>518.9*	26	166	10	18.438
		562.95>269	26	166	14	18.438
PFDoA	LT	612.95>568.9*	26	166	10	19.005
		612.95>319	26	166	18	19.005
PFTrDA	LT	662.95>618.9*	26	166	10	19.490
		662.95>168.9	26	166	34	19.490
PFTeDA	LT	712.94>669*	26	166	10	19.912
		712.94>169	26	166	30	19.912
		**Perfluoroalkyl sulfonic acids (PFSAs)**				
PFBS	HT	298.94>80*	53	166	38	11.735
		298.94>99	53	166	30	11.735
PFPeS	HT	348.94>80*	61	166	46	13.724
		348.94>99	61	166	42	13.724

PFHxS	HT	398.59>80*	15	166	42	15.080
		398.59>99	15	166	42	15.080
PFHpS	HT	448.93>79.9*	15	166	46	16.125
		448.93>98.8	15	166	46	16.125
PFOS	HT	498.93>80*	15	166	58	16.999
		498.93>229.9	15	166	46	16.999
		498.93>129.9	15	166	50	16.999
		498.93>99	15	166	42	16.999
PFNS	HT	548.92>80*	14	166	54	17.734
		548.92>99	14	166	50	17.734
PFDS	HT	598.92>80*	14	166	58	18.363
		598.92>99	14	166	54	18.363
**Per and polyfluoroalkyl ether carboxylic acids (PFECAs)**						
PFMOAA	LT	178.97>85.1*	43	166	10	2.728
		178.97>135	43	166	0	2.728
PMPA	LT	185>119*	43	166	10	7.001
		229>85	43	166	15	7.001
PEPA	LT	235>135*	43	166	0	11.788
		279>235	43	166	16	11.788
PFPrOPrA	LT	284.59>169*	73	166	10	14.023
		284.59>184.9	73	166	14	14.023
PFO2HxA	LT	244.9+7>85.1*	43	166	10	10.066
		244.97>151	43	166	5	10.066
PFO3OA	LT	310.96>85	73	166	10	13.762
PFO4DA	LT	376.95>84.9*	17	166	10	15.610
		376.95>151	17	166	5	15.610
PFO5DoA	LT	442.94>85.1*	19	166	18	16.854
		442.94>150	19	166	5	16.854
HydroEVE	LT	426.96>283*	17	166	10	15.107
		426.96>213	17	166	30	15.107
ADONA	LT	376.96>250.9*	17	166	10	15.215

		376.96>85	17	166	30	15.215
**Per and polyfluoroalkyl ether sulfonic acid (PFESAs)**						
Nafion by-product 2	HT	462.93>263*	15	166	26	15.125
		462.93>213	15	166	38	15.125
NVHOS	HT	296.94>80.1*	53	166	42	9.135
		296.94>116.9	53	166	30	9.135
F-53B major	LT	530.9>350.9*	19	166	30	17.481
		530.9>83.1	19	166	34	17.481
**Diprotic ether acid**						
Nafion by-product 4	HT	440.93>196.9*	53	166	34	7.698
		440.93>240.8	53	166	26	7.698
**Perfluoroalkane sulfonamido acetic acids (FASAAs)**						
N-MeFOSAA	HT	569.96>418.8*	14	166	22	18.095
		569.96>511.8	14	166	22	18.095
N-EtFOSAA	HT	583.98>419*	14	166	18	18.434
		583.98>526	14	166	18	18.434
**Perfluoroalkane sulfonamides (FASAs)**						
PFBSA	HT	297.96>77.9*	61	166	25	13.569
		297.96>119.05	61	166	18	13.569
PFHxSA	HT	397.95>77.9*	15	166	30	16.662
		397.95>168.8	15	166	30	16.662
PFOSA	HT	497.94>78.1*	14	166	38	18.437
		497.94>48.1	14	166	60	18.437
**Fluorotelomer sulfonic acids (FTSs)**						
4:2 FTS	HT	326.97>307*	61	166	18	13.284
		326.97>81	61	166	30	13.284
6:2 FTS	HT	426.97>407*	15	166	26	16.022

		426.97>81	15	166	38	16.022
8:2 FTS	HT	526.96>506.9*	14	166	30	17.748
		526.96>81.1	14	166	34	17.748
**Fluorotelomer carboxylic acids (FTCAs)**						
6:2 FTCA	LT	376.98>293*	17	166	18	15.369
		376.98>63.1	17	166	10	15.369
8:2 FTCA	LT	476.98>392.9*	19	166	10	17.338
		476.98>63.1	19	166	14	17.338
6:2 FTUCA	LT	356.98>293*	17	166	14	15.288
		356.98>242.8	17	166	38	15.288
8:2 FTUCA	LT	456.97>392.8*	19	166	10	17.291
		456.97>342.9	19	166	46	17.291
**Isotopically Labelled Internal Standards**						
MPFBA	LT	216.99>172	43	166	10	5.834
M5PFPeA	LT	267.99>222.9	43	166	10	10.966
M5PFHxA	LT	318>273	73	166	10	13.483
M4PFHpA	LT	366.98>322	17	166	10	15.006
M8PFOA	LT	420.99>376	17	166	10	16.127
M9PFNA	LT	472>427	19	166	10	17.033
M6PFDA	LT	519>474	19	166	10	17.796
M7PFUnDA	LT	569.98>525	26	166	10	18.438
MPFDoA	LT	614.96>569.9	26	166	10	19.005
M2PFTeDA	LT	714.95>669.9	26	166	10	19.912
M3PFBS	HT	301.95>80	53	166	38	11.735
MPFHxS	HT	401.9>79.9	15	166	38	15.080
M8PFOS	HT	507>99	15	166	42	16.999
M3HFPO-DA	LT	287>169	73	166	10	14.023
d3N-MeFOSAA	HT	572.98>419	14	166	18	18.095

d5N-EtFOSAA	HT	588.99>418.9	14	166	18	18.434
M8PFOSA	HT	505.97>78	14	166	38	18.437
M4:2FTS	HT	328.98>309	61	166	22	13.284
M6:2FTS	HT	428.97>409	15	166	26	16.022
M8:2FTS	HT	528.97>508.9	14	166	30	17.748
M6:2 FTCA	LT	378.99>294	17	166	22	15.369
M8:2 FTCA	LT	478.98>394	19	166	18	17.338
M6:2 FTUCA	LT	358.98>294	17	166	10	15.288
M8:2 FTUCA	LT	458.98>394	19	166	14	17.291

* Denotes quantitation ion for analytes with more than one mass transition

[a] Source temperature: 400°C for HT; 250°C for LT

Table S6. PFAS spike-recovery in different matrices

PFAS	MRL (ng/g)	PFAS spike-recovery in different matrices (%)										
		Blackberry	Blueberry	Corn	Fig	Okra	Peach	Peas	Pecan	Pickled green beans	Squash	Tomato
PFMOAA	0.05	168	233	66	83	35	124	48	98	46	54	83
PMPA	0.025	85	132	97	102	102	125	67	101	96	86	92
PEPA	0.01	139	153	74	117	112	100	82	73	73	144	93
GenX	0.01	92	92	89	86	91	85	77	76	86	84	85
PFO2HxA	0.01	113	110	96	82	34	38	58	76	88	98	68
PFO3OA	0.01	91	74	46	36	75	37	73	79	42	40	28
PFO4DA	0.01	88	99	143	93	95	111	89	87	92	115	81
PFO5DoA	0.01	138	87	104	76	83	58	80	58	104	107	81
Adona	0.01	86	97	141	91	93	109	89	85	91	112	79
HydroEVE	0.01	148	112	163	94	93	94	96	89	94	103	92
NVHOS	0.01	55	107	105	90	90	78	163	86	99	88	92
Nafion BP2	0.01	220	100	98	88	94	92	101	92	94	93	93
F53bMajor	0.01	172	119	181	117	85	108	59	69	120	133	97
PFBA	0.025	95	88	87	95	92	112	69	82	90	NA	96
PFPeA	0.01	97	92	84	81	92	89	83	81	91	90	90
PFHxA	0.01	91	94	86	87	91	89	82	82	89	89	91
PFHpA	0.01	93	93	80	88	94	88	82	82	90	89	88
PFOA	0.01	92	93	85	88	96	88	82	80	91	89	90
PFNA	0.01	92	93	82	85	95	89	83	83	89	92	89
PFDA	0.01	94	95	82	88	94	88	83	82	90	90	88
PFUnDA	0.01	93	95	83	91	96	90	85	83	93	92	90
PFDoDA	0.01	96	97	78	92	99	92	78	78	96	93	91

PFTrDA	0.10	123	85	150	72	232	86	503	186	78	51	62
PFTeDA	0.10	91	75	86	91	96	82	88	87	100	95	94
PFBS	0.010	95	95	83	88	94	90	83	82	91	92	90
PFPeS	0.025	58	56	40	32	55	36	34	31	44	33	99
PFHxS	0.01	133	100	85	90	97	92	82	85	92	93	91
PFHpS	0.01	44	43	64	41	47	35	227	60	53	41	36
PFOS	0.01	105	97	82	86	94	92	81	79	89	89	89
PFNS	0.025	70	100	89	105	117	93	67	84	129	110	114
PFDS	0.05	187	169	253	266	174	241	151	247	212	292	178
42FTS	0.01	100	101	89	120	111	96	80	81	96	89	111
62FTS	0.01	98	100	75	93	101	94	77	77	96	94	96
82FTS	0.01	98	99	78	93	99	93	79	78	97	95	96
62FTCA	0.01	96	97	55	90	89	91	58	57	91	88	89
82FTCA	0.025	97	96	49	92	99	90	42	49	93	92	92
62FTUCA	0.025	93	92	19	86	95	86	7	7	89	89	86
82FTUCA	0.025	94	95	6	88	95	89	5	6	91	90	89
EtFOSAA	0.01	99	95	72	89	91	94	81	71	90	92	90
MeFOSAA	0.025	92	103	91	93	126	106	95	69	117	103	111
PFBSA	0.05	54	52	29	44	70	54	59	56	60	24	119
PFHxSA	0.05	44	36	133	167	190	44	502	124	83	125	95
PFOSA	0.025	100	106	78	92	96	92	83	87	97	92	94

Table S7. PFAS concentrations in groundwater samples

PFAS	MRL (ng/L)	PFAS concentration (ng/L)				
		A	B	C	D	E
PFMOAA	5	50	4	<MRL	9	<MRL
PMPA	10	439	45	16	82	26
PEPA	2	159	10	2	16	4
GenX	0.5	304	24	3	23	<MRL
PFO2HxA	2	172	17	<MRL	24	12
PFO3OA	1	28	3	<MRL	1	1
PFO4DA	0.5	9	<MRL	<MRL	<MRL	<MRL
Hydro-Eve	0.5	7	<MRL	<MRL	<MRL	<MRL
NVHOS	1	5	<MRL	<MRL	<MRL	1
Nafion by product 2	1	25	6	<MRL	<MRL	2
PFBA	5	3	<MRL	<MRL	<MRL	2
PFPeA	1	5	<MRL	<MRL	1	8
PFHxA	0.5	2	<MRL	<MRL	<MRL	6
PFHpA	0.5	1	<MRL	<MRL	<MRL	3
PFOA	1	1	<MRL	2	3	8
PFBS	0.5	<MRL	6	<MRL	<MRL	<MRL
PFOS	5	<MRL	<MRL	<MRL	<MRL	33
Sum	/	1209	115	23	159	106

Table S8. Statistics of PFAS concentration in residential garden produce samples (n=53)

**PFAS**	**Detection frequency (%)**	**Percentile concentration (ng/g wet weight)**					
		**mean**	**min**	**25%**	**50%**	**75%**	**max**
PFMOAA	81	0.867	<MRL	0.052	0.295	0.866	11.469
PMPA	96	2.074	<MRL	0.293	0.755	2.267	26.351
PEPA	92	0.231	<MRL	0.027	0.146	0.256	2.680
GenX	72	0.141	<MRL	<MRL	0.044	0.146	1.024

PFO2HxA	100	1.190	0.016	0.081	0.352	1.198	9.557
PFO3OA	47	0.025	<MRL	<MRL	<MRL	0.028	0.178
PFO4DA	0	<MRL	<MRL	<MRL	<MRL	<MRL	<MRL
PFO5DoA	0	<MRL	<MRL	<MRL	<MRL	<MRL	<MRL
Adona	0	<MRL	<MRL	<MRL	<MRL	<MRL	<MRL
HydroEVE	0	<MRL	<MRL	<MRL	<MRL	<MRL	<MRL
NVHOS	17	0.008	<MRL	<MRL	<MRL	<MRL	0.267
Nafion BP2	13	0.003	<MRL	<MRL	<MRL	<MRL	0.032
F53bMajor	0	<MRL	<MRL	<MRL	<MRL	<MRL	<MRL
PFPeA	43	0.016	<MRL	<MRL	<MRL	0.025	0.096
PFHxA	19	0.018	<MRL	<MRL	<MRL	<MRL	0.583
PFHpA	0	<MRL	<MRL	<MRL	<MRL	<MRL	<MRL
PFOA	6	0.003	<MRL	<MRL	<MRL	<MRL	0.103
PFNA	0	<MRL	<MRL	<MRL	<MRL	<MRL	<MRL
PFDA	0	<MRL	<MRL	<MRL	<MRL	<MRL	<MRL
PFUnDA	0	<MRL	<MRL	<MRL	<MRL	<MRL	<MRL
PFDoDA	0	<MRL	<MRL	<MRL	<MRL	<MRL	<MRL
PFTrDA	0	<MRL	<MRL	<MRL	<MRL	<MRL	<MRL
PFTeDA	0	<MRL	<MRL	<MRL	<MRL	<MRL	<MRL
PFBS	6	0.001	<MRL	<MRL	<MRL	<MRL	0.020
PFPeS	0	<MRL	<MRL	<MRL	<MRL	<MRL	<MRL
PFHxS	2	0.001	<MRL	<MRL	<MRL	<MRL	0.049
PFHpS	0	<MRL	<MRL	<MRL	<MRL	<MRL	<MRL
PFOS	0	<MRL	<MRL	<MRL	<MRL	<MRL	<MRL
PFNS	0	<MRL	<MRL	<MRL	<MRL	<MRL	<MRL
PFDS	2	0.007	<MRL	<MRL	<MRL	<MRL	0.345
42FTS	0	<MRL	<MRL	<MRL	<MRL	<MRL	<MRL
62FTS	8	0.005	<MRL	<MRL	<MRL	<MRL	0.106
82FTS	0	<MRL	<MRL	<MRL	<MRL	<MRL	<MRL
62FTCA	0	<MRL	<MRL	<MRL	<MRL	<MRL	<MRL
82FTCA	0	<MRL	<MRL	<MRL	<MRL	<MRL	<MRL

62FTUCA	0	<MRL	<MRL	<MRL	<MRL	<MRL	<MRL
82FTUCA	0	<MRL	<MRL	<MRL	<MRL	<MRL	<MRL
EtFOSAA	2	0.001	<MRL	<MRL	<MRL	<MRL	0.040
MeFOSAA	0	<MRL	<MRL	<MRL	<MRL	<MRL	<MRL
PFBSA	2	0.001	<MRL	<MRL	<MRL	<MRL	0.040
PFHxSA	0	<MRL	<MRL	<MRL	<MRL	<MRL	<MRL
PFOSA	6	0.002	<MRL	<MRL	<MRL	<MRL	0.036

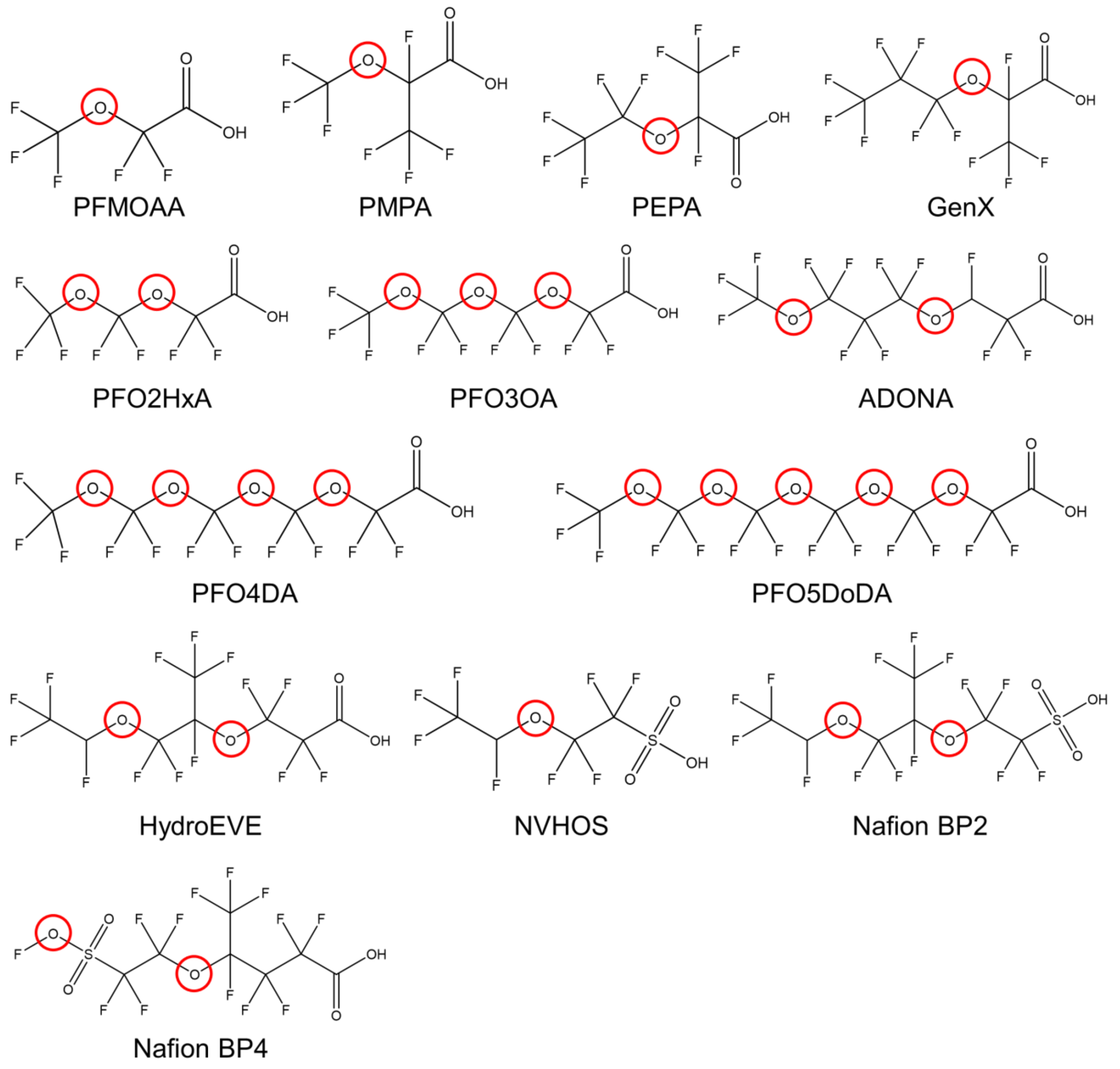

Figure S1. PFEAs detected in private wells and public water systems in North Carolina

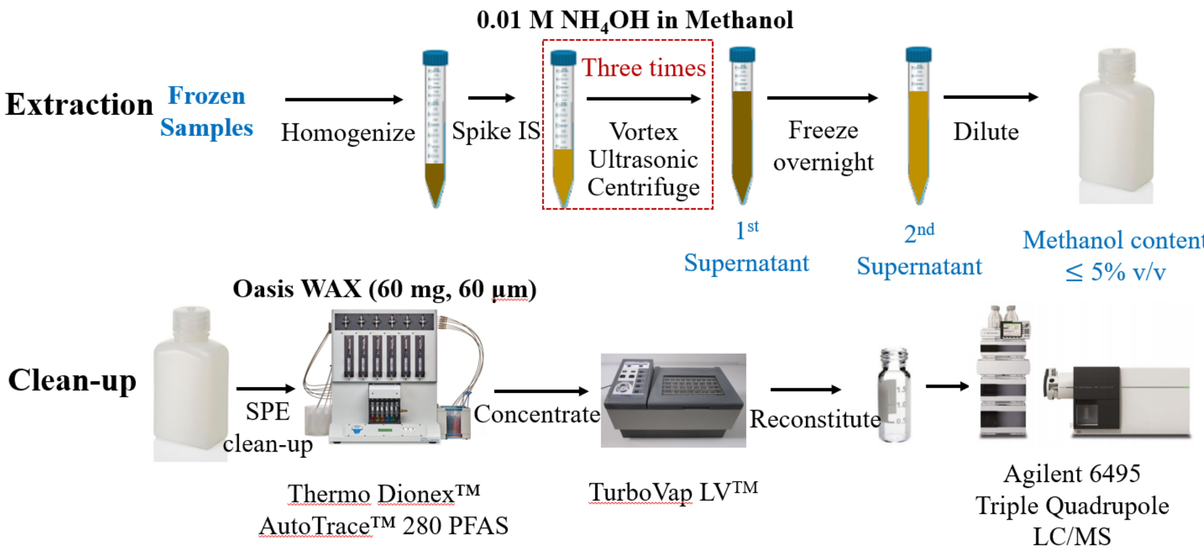

Figure S2. Extraction workflow

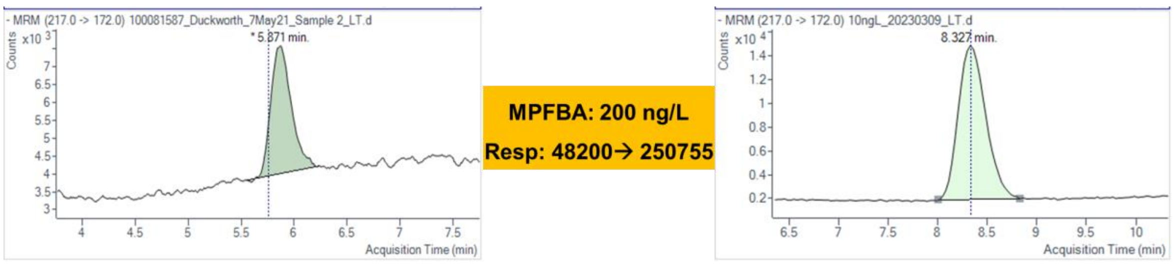

Figure S3. Response of MPFBA peaks before and after method modification. The retention time shift (from 5.871 min to 8.327 min) was due to a change in the mobile phase flow rate (from 0.7 mL/min to 0.5 mL/min) to address system backpressure concerns. Other LC and MS parameters were kept consistent between the two methods.

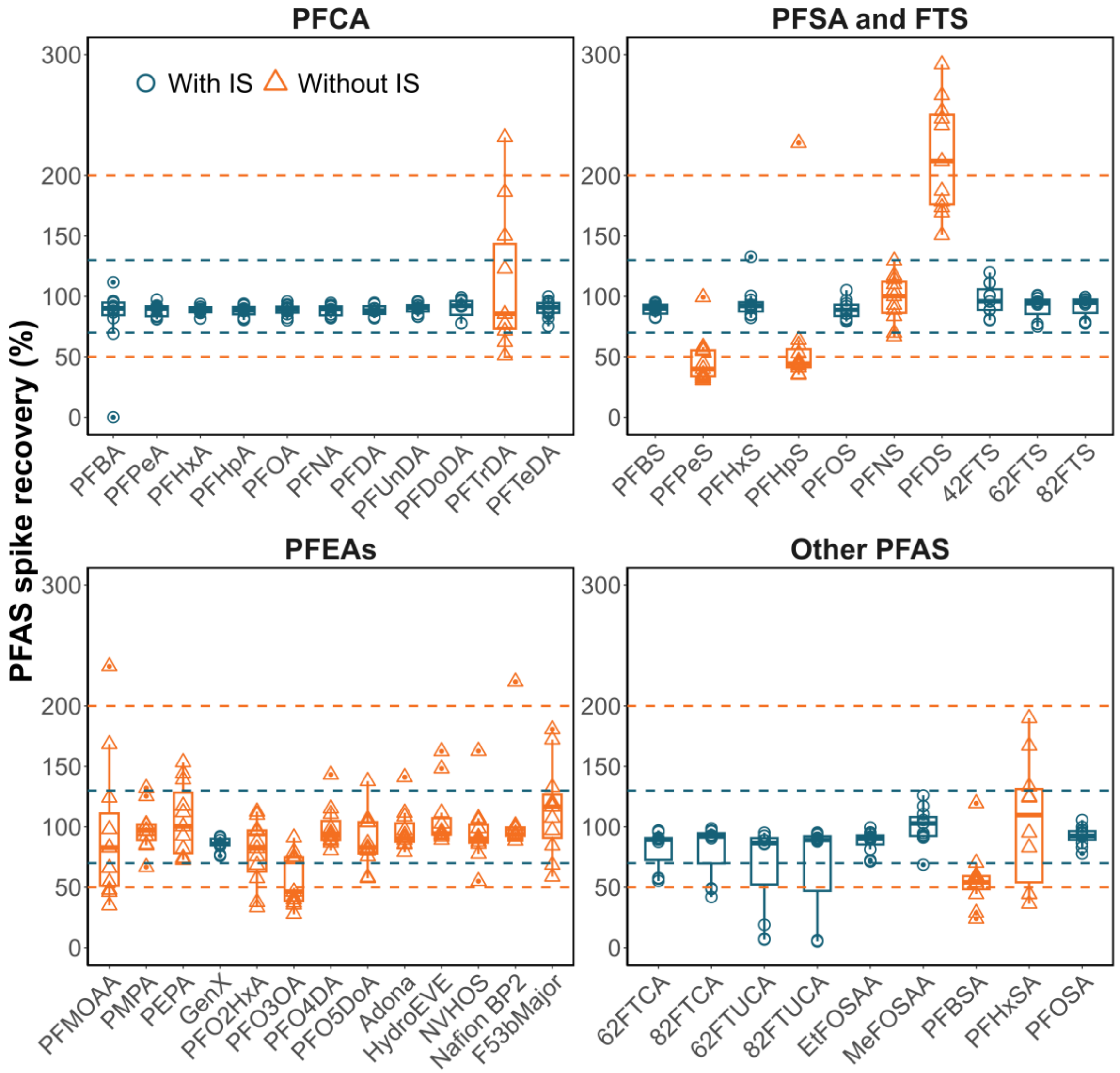

Figure S4. PFAS spike-recovery in different matrices

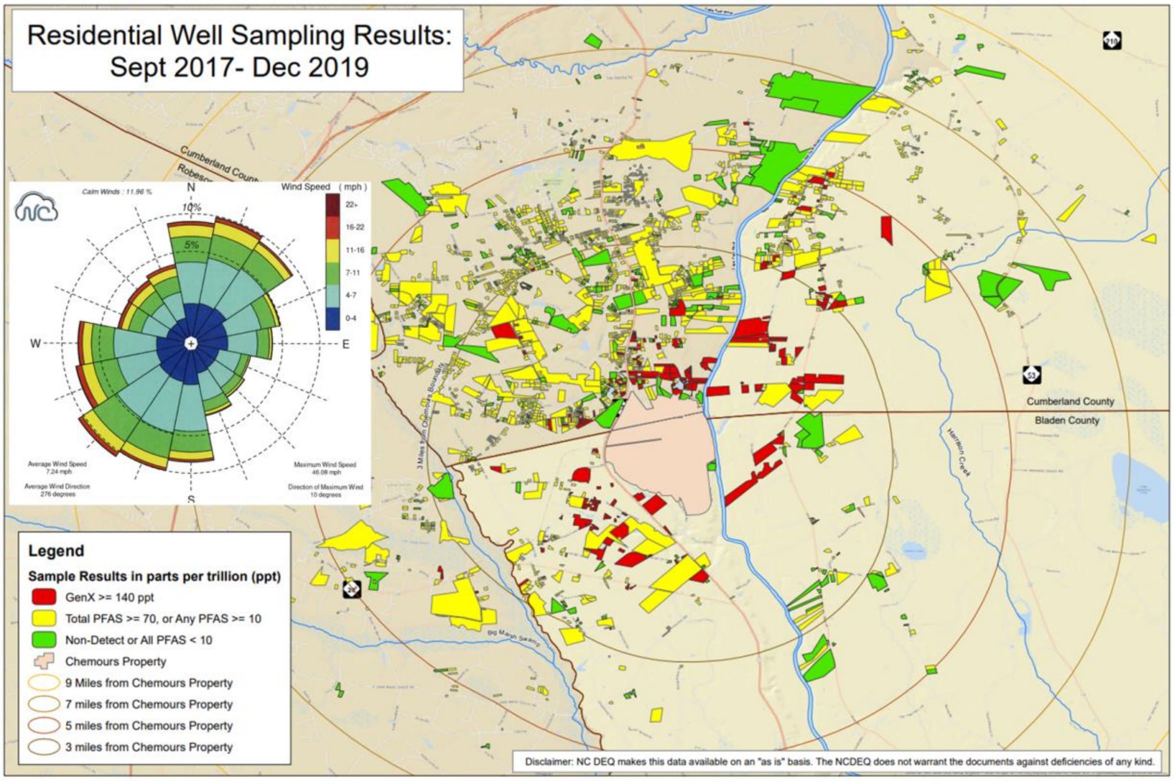

Figure S5. NCDEQ and Chemours residential well sampling results (09/2017-12/2019)[1] with Wind Rose (01/1998-09/2019) for Fayetteville Airport (10 miles North of the circled area)[2]

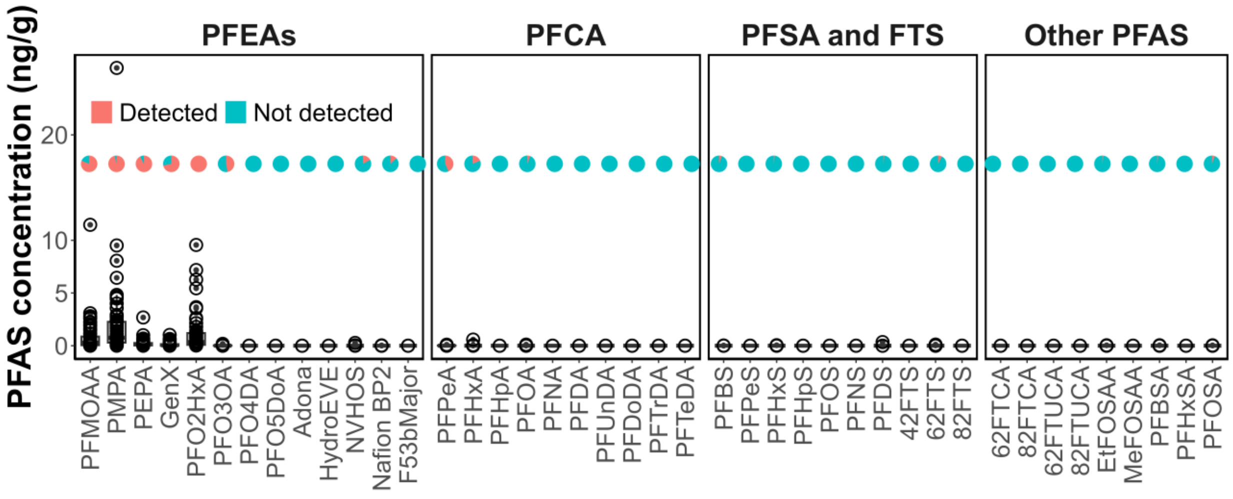

Figure S6. Concentrations and detection frequencies of PFASs in 53 produce samples collected in an impacted community near a fluorochemical manufacturer in Fayetteville, North Carolina.

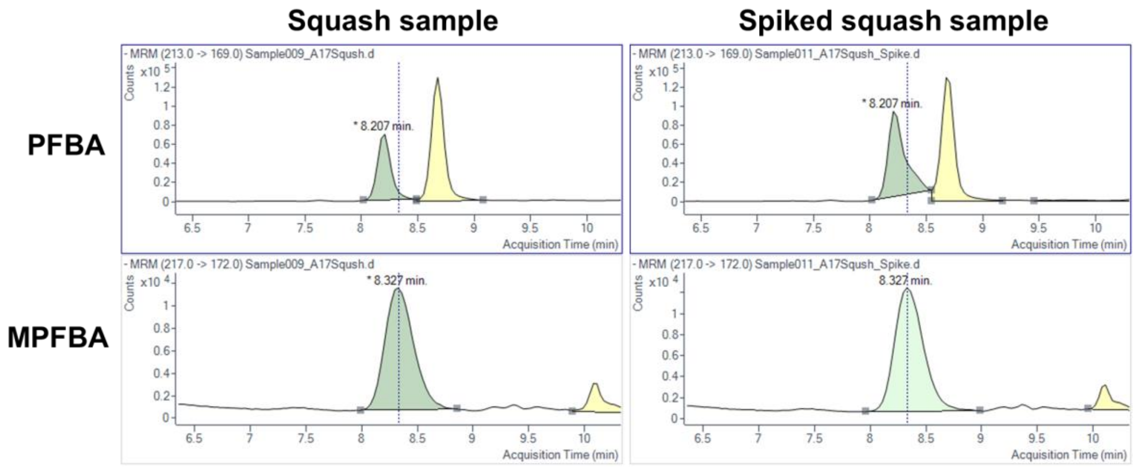

Figure S7. Interference peak observed for PFBA in squash matrix (left: original sample; right: matrix-spike sample with 200 ng/L PFBA)

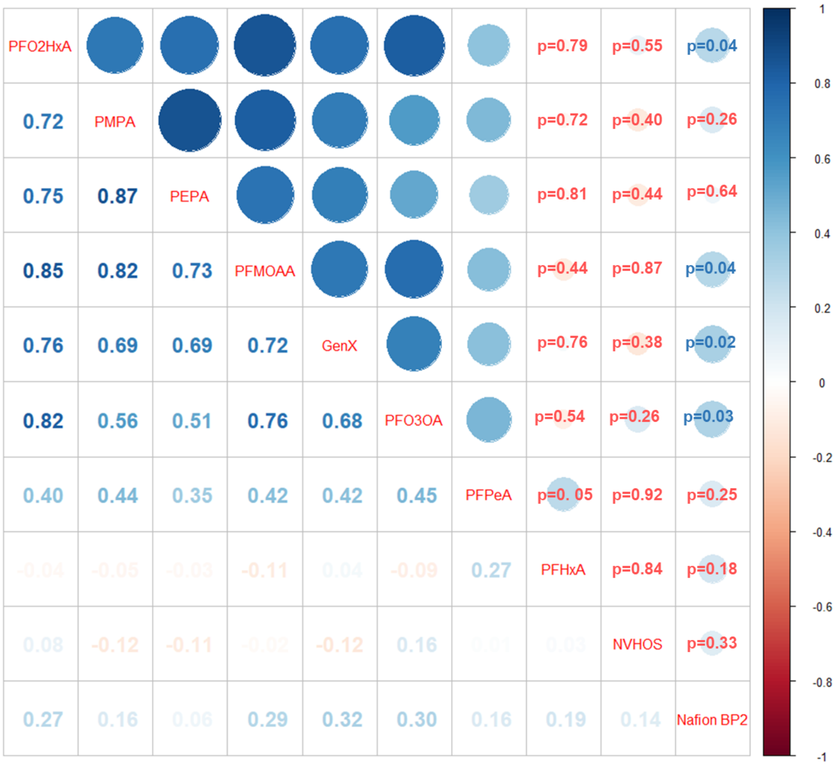

Figure S8. Spearman correlation between different PFAS in produce. The color scale shows the range of Spearman correlation coefficients, and coefficients are shown below the diagonal. The size of circles above the diagonal represents the significance of the correlation coefficients (larger circles correspond to lower p values). When p values were <0.01, circles were not labeled.

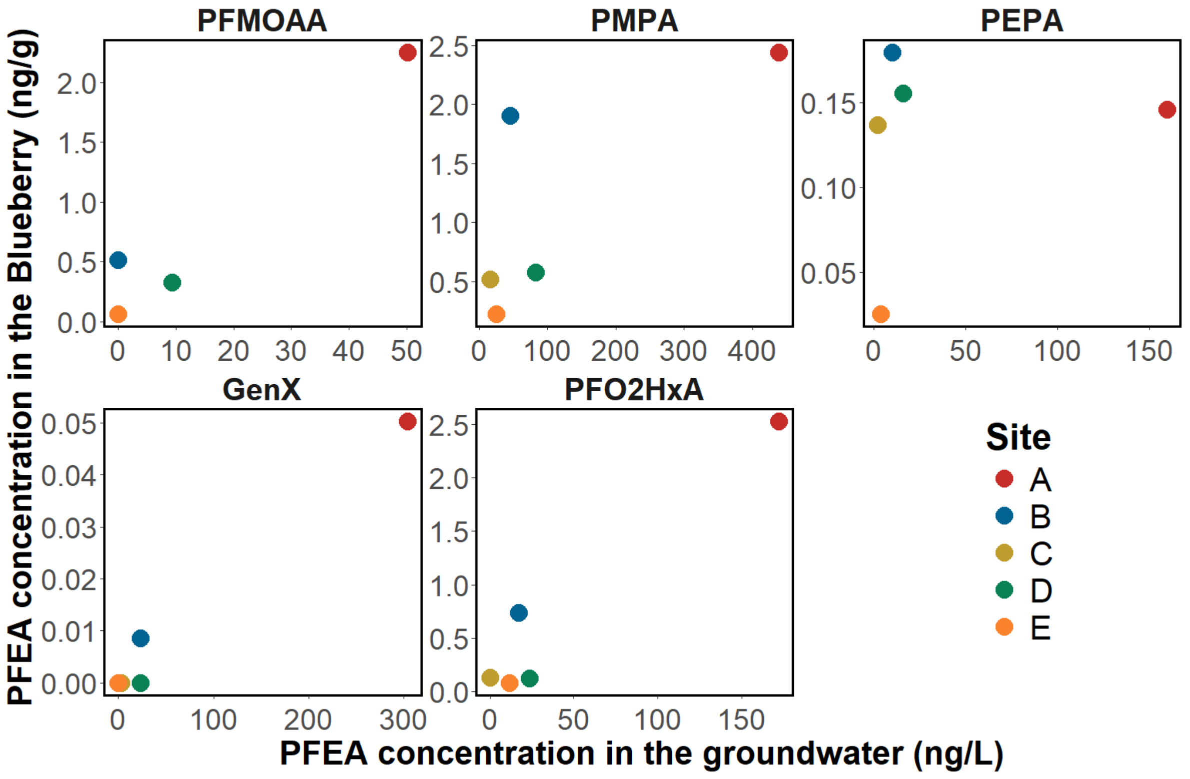

Figure S9. Correlation between PFAS concentrations in groundwater and in blueberry samples

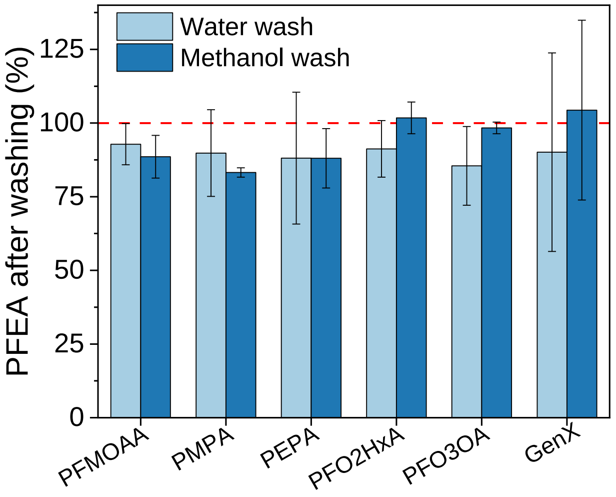

Figure S10. Effect of washing on PFAS concentrations in blueberries. The bars represent the mean percentage of PFEAs remaining in the blueberry samples, and whiskers indicate one standard deviation.

References

1. North Carolina Department of Environmental Quality (NC DEQ), PFAS residential well sampling Fayetteville region results results through 12/2022. https://files.nc.gov/deq/images/2023-03/Q4%20Fay%20Residential%20Well%20Sampling.png?VersionId=X2gMJ8To3rfw5IrSJELCD4UwWy..dwdq (accessed September 6, 2024)
2. North Carolina Department of Environmental Quality (NC DEQ), Wind Rose for Fayetteville Airport (KFAY). **2019**, https://files.nc.gov/ncdeq/GenX/consentorder/paragraph12/Wind_Rose.pdf (accessed September 6, 2024)
